# Correction: Structural basis for substrate recognition and inhibition of thioredoxin glutathione reductase from *Schistosoma japonicum*: Implications for antiparasitic development

**DOI:** 10.1371/journal.ppat.1014386

**Published:** 2026-06-30

**Authors:** Songqing Wang, Wenbin Hong, Shukun Zhong, Zhijian Liang, Tianyichen Xiao, Chuchu Zhang, Xianshu Liu, Ziyi Dai, Yunlong Li, Siqi Wu, Qixu Cai, Caiming Wu, Yuxuan Huang, Peicheng Hong, Haixia Ren, Shaowei Li, Tianwei Lin, Xueqin Chen, Shuaiqin Huang

The authors, Songqing Wang, Wenbin Hong and Shukun Zhong, should be noted as shared first co-authors on this work.
