# Supplementary figures and images for: Structural basis for substrate recognition and inhibition of thioredoxin glutathione reductase from Schistosoma japonicum: Implications for antiparasitic development
Source: PLoS Pathog. 2026 Apr 24;22(4):e1014125. doi: 10.1371/journal.ppat.1014125 (PMC13138743; doi:10.1371/journal.ppat.1014125)

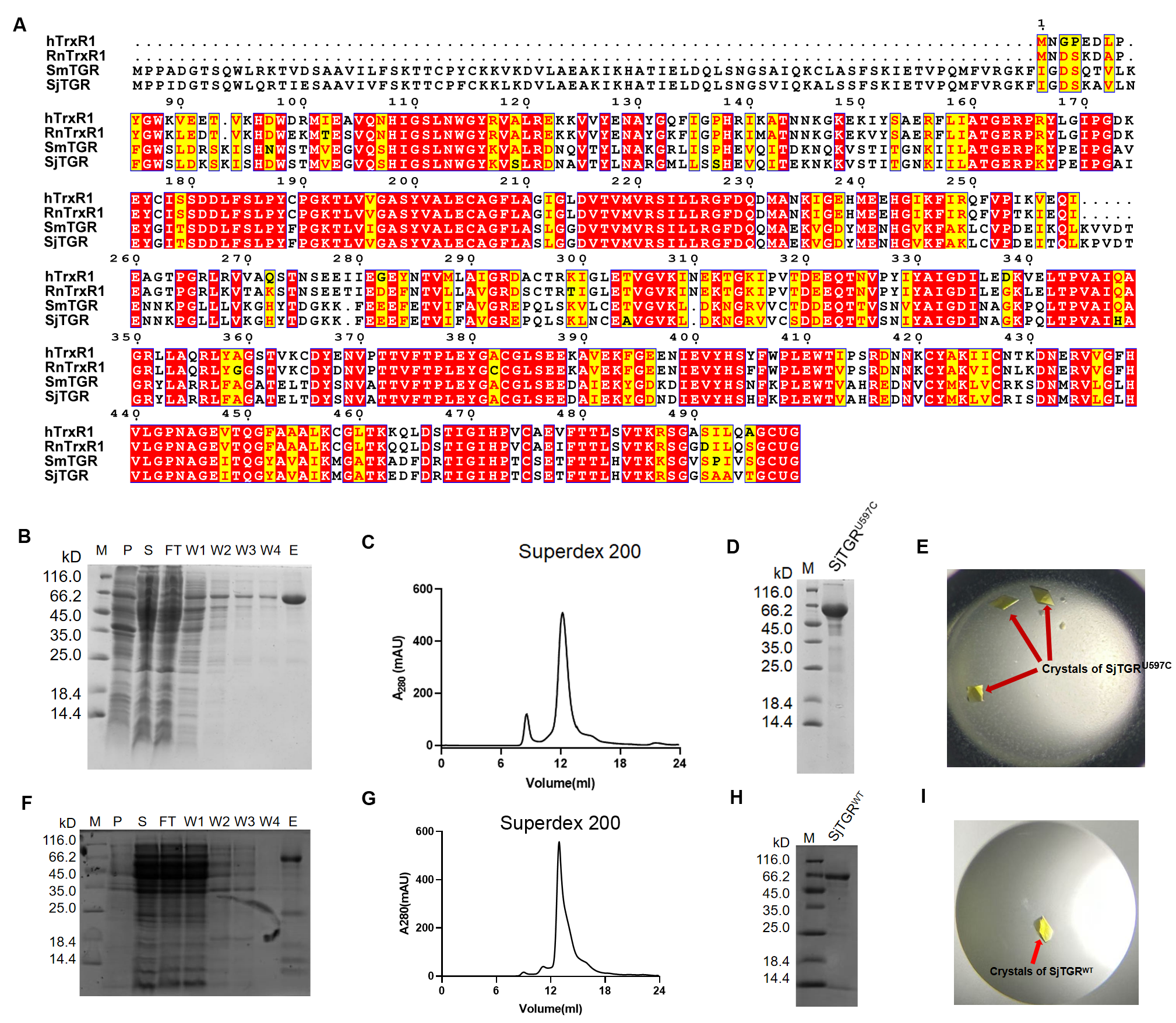

Supplement: S1 Fig — (A) Sequence alignment of thioredoxin reductases from Homo sapiens (hTrxR1, NCBI accession: NP_877393.1), Rattus norvegicus (RnTrxR1, NCBI accession: O89049.5), Schistosoma mansoni (SmTGR, NCBI accession: XP_018649018.1), and Schistosoma japonicum (SjTGR, NCBI accession: ACH86016.1). (B) 15% SDS-PAGE analysis of SjTGRU597C purification by the Ni-NTA affinity chromatography (M: marker, P: precipitate, S: supernatant, FT: flow-through, W1–4: wash fractions 1–4, E: eluate). (C) Elution profile of SjTGRU597C from Superdex 200 Increase 10/300 GL size-exclusion chromatography. (D) Gel filtration chromatography reveals the purified SjTGRU597C protein, which was initially obtained through Ni beads affinity chromatography and subsequently purified further using Superdex 200 size-exclusion filtration. (E) Crystals of the SjTGRU597C protein. (F) 15% SDS-PAGE analysis of SjTGRWT purification by the Ni-NTA affinity chromatography (M: marker, P: precipitate, S: supernatant, FT: flow-through, W1–4: wash fractions 1–4, E: eluate). (G) Elution profile of SjTGRWT from Superdex 200 Increase 10/300 GL size-exclusion chromatography. (H) Gel filtration chromatography reveals the purified SjTGRWT protein, which was initially obtained through Ni beads affinity chromatography and subsequently purified further using Superdex 200 size-exclusion filtration. (E) Crystals of the SjTGRWT protein. (TIF) [file ppat.1014125.s001.tif]

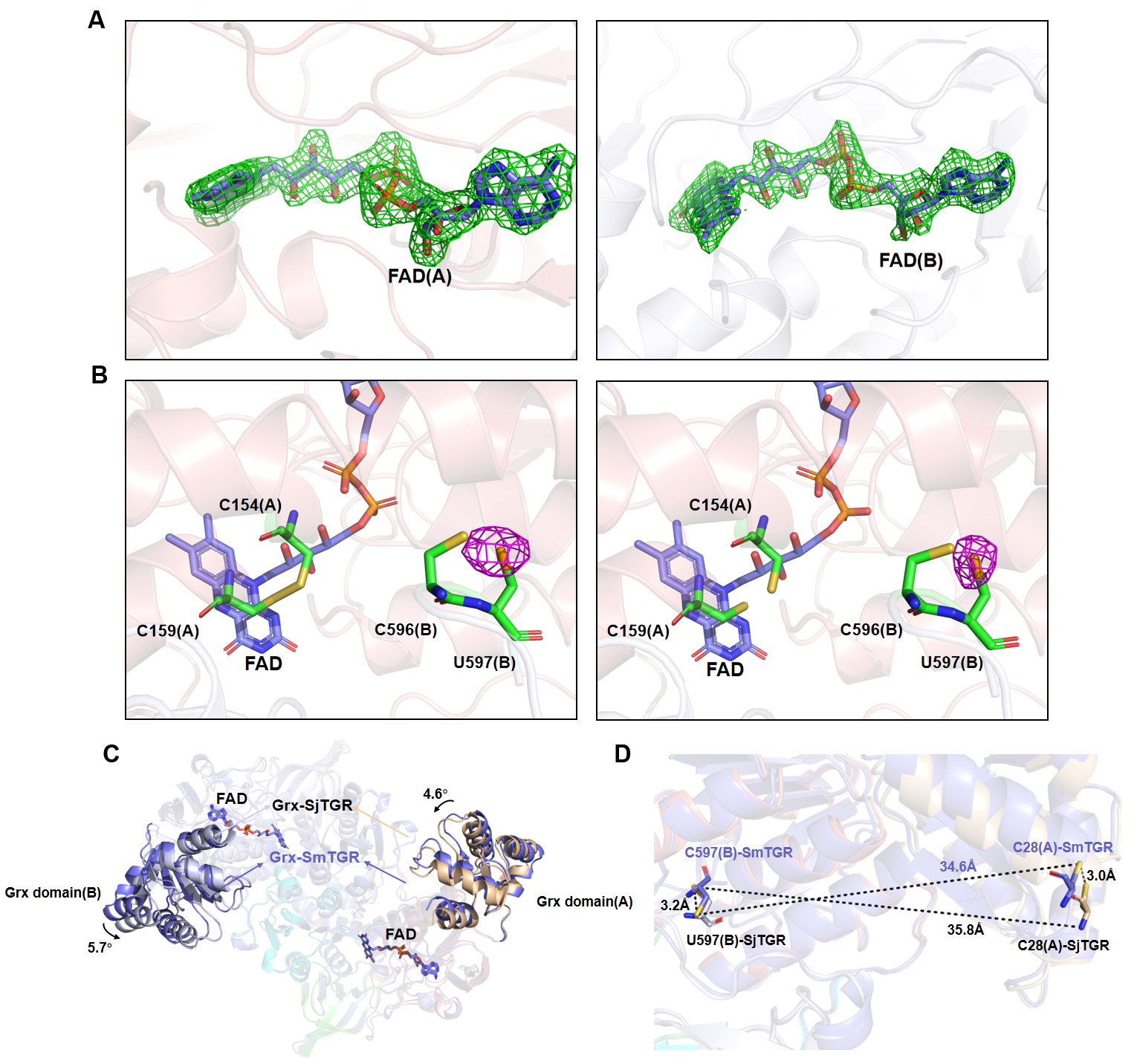

Supplement: S2 Fig — (A) The FAD Fo-Fc electron density map for subunit A and subunit B of SjTGR (PDB: 9LWM), contoured at 3σ and displayed in green meshes. (B) The anomalous scattering electron density of selenium (Se), contoured at 5σ in violet meshes (PDB: 9LWM and 22EY). (C) Structural superposition of SmTGR (blue) (PDB: 7B02) and SjTGR (PDB: 9LWM) TrxR domains was performed, with both TrxR domains rendered transparent to accentuate divergent orientations of their Grx domains. (D) Electron transfer path between the Grx domain redox center (C28-C31) and the adjacent subunit’s C-terminal selenothiol motif (C596-U597) in SmTGR (PDB: 7B02) and SjTGR (PDB: 9LWM). Distance measurements are shown as black dashed lines. (TIF) [file ppat.1014125.s002.tif]

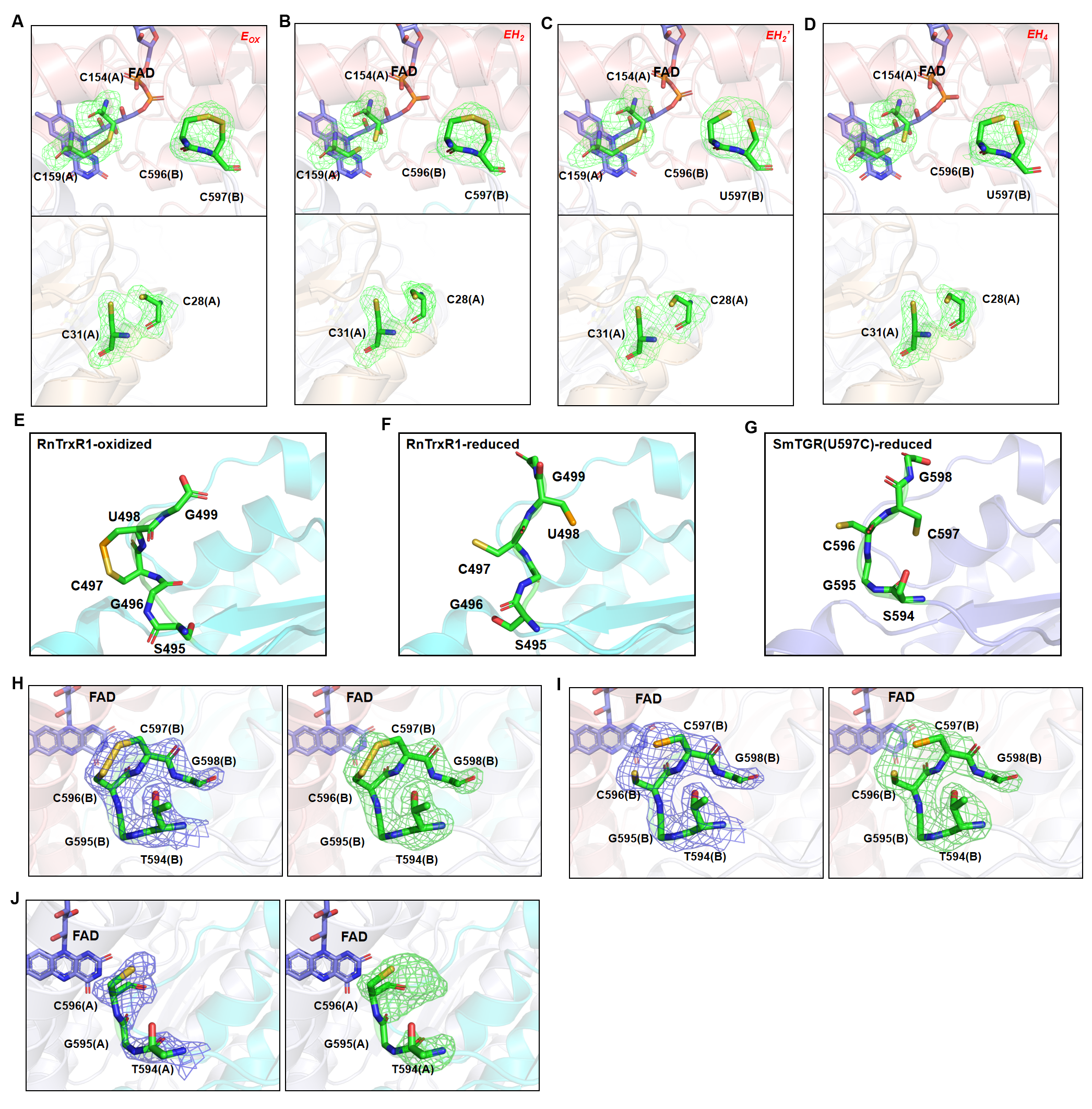

Supplement: S3 Fig — (A-D) The Fo-Fc electron density maps for the three redox centers of SjTGR-WT (GCUG) and SjTGR-U597C (GCCG) with different redox states are shown, contoured at 3σ in green meshes. The corresponding PDB entries are 9LWZ (A), 22FC (B), 22EY (C), and 9LWM (D). (E-F) The conformations of the last five residues (green sticks) of RnTrxR1 in the oxidized state (E) (PDB: 3EAO) and the reduced state (F) (PDB: 3EAN). (G) The conformations of the residues (green sticks) at the C-terminal of SmTGR (PDB: 7B02) in the reduced state. (H-I) The 2Fo-Fc electron density maps (1σ, blue meshes) and the Fo-Fc electron density maps (3σ, green meshes) of SjTGR subunit B C-terminal in the oxidized state (PDB: 22FC) and the reduced state (PDB: 22EY). (J) The 2Fo-Fc electron density map (1σ, blue meshes) and the Fo-Fc electron density map (3σ, green meshes) of the C-terminal of SjTGR (PDB: 22FC) subunit A. (TIF) [file ppat.1014125.s003.tif]

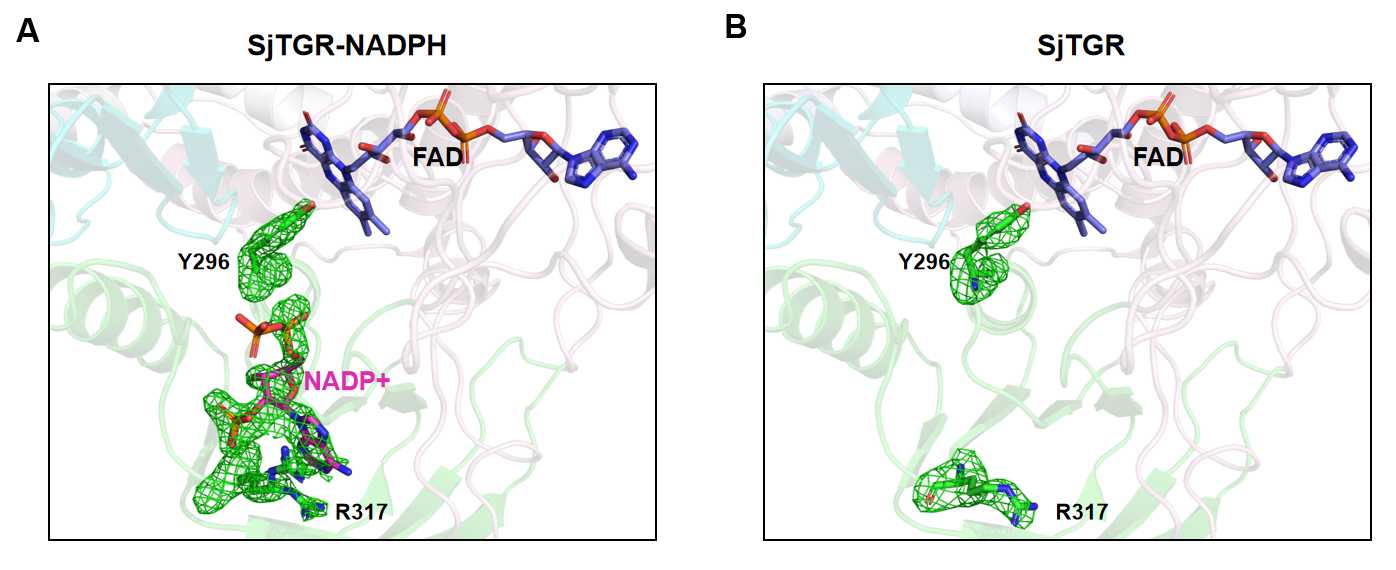

Supplement: S4 Fig — Y296 and R317 (green sticks) within SjTGR are shown, contoured at 3σ in green meshes. In panel (A), the NADPH-bound conformation of SjTGR (PDB: 22FD) is illustrated, whereas panel (B) displays the unbound state (PDB: 22EY). (TIF) [file ppat.1014125.s004.tif]

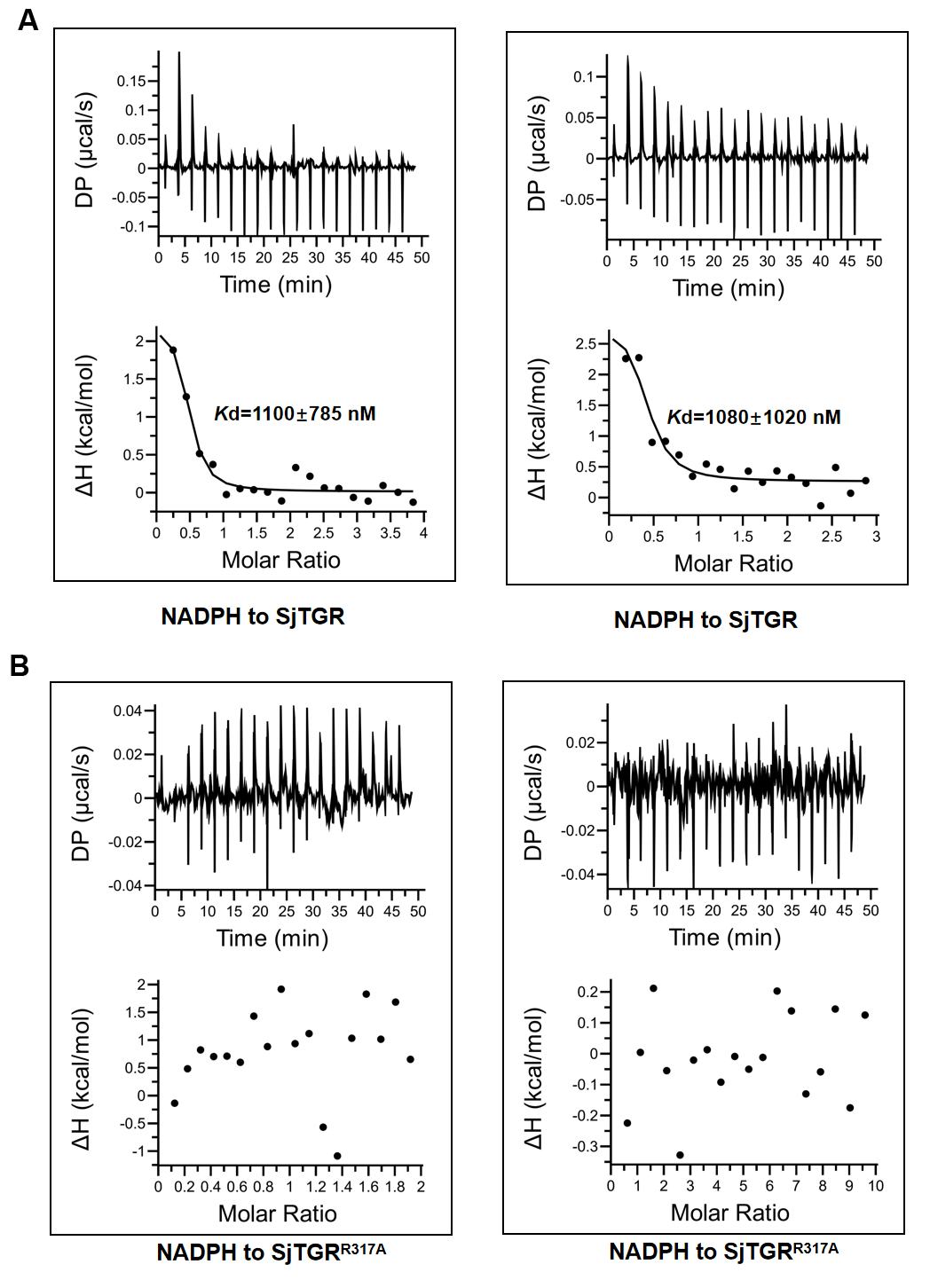

Supplement: S5 Fig — (A) The two additional technical replicates for ITC analysis of NADPH binding to wild-type SjTGR. (B) The two additional technical replicates for ITC analysis of NADPH binding to SjTGRR317A. (TIF) [file ppat.1014125.s005.tif]

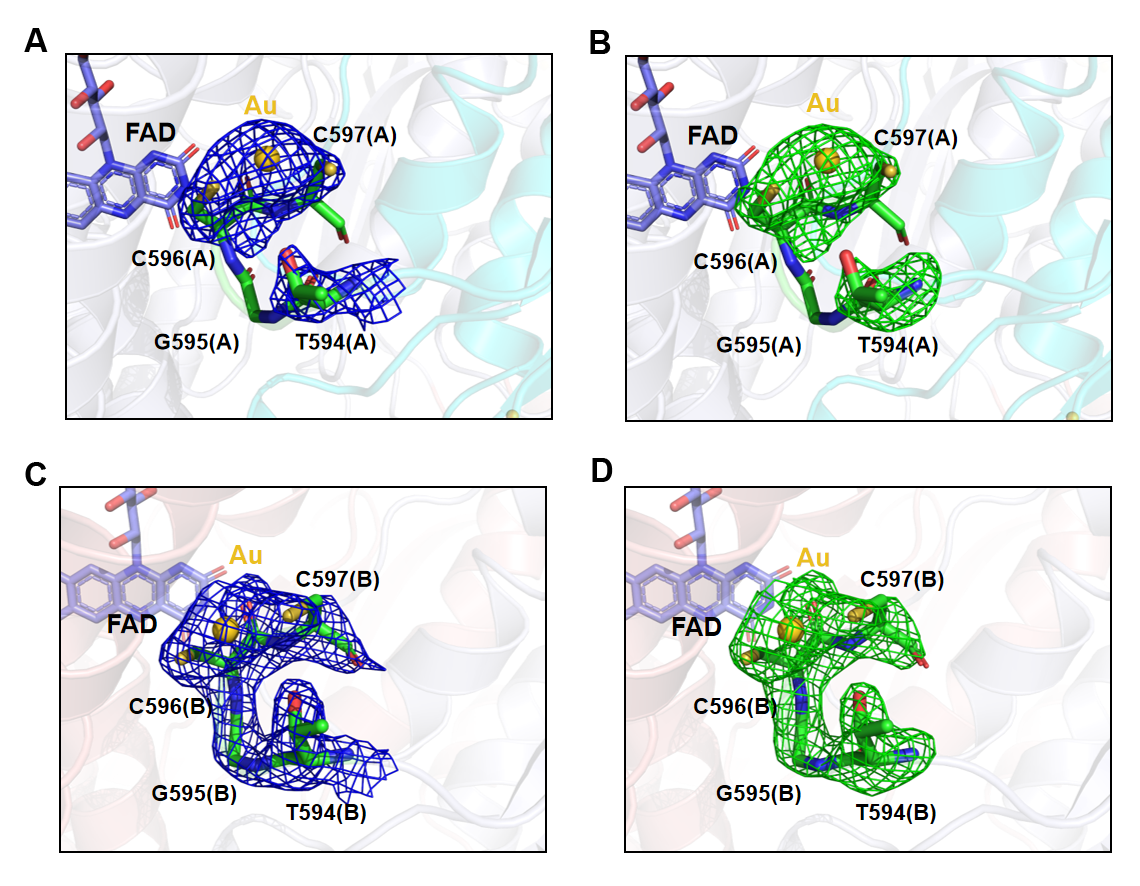

Supplement: S6 Fig — (A-B) The 2Fo-Fc electron density map (1σ, blue meshes) and the Fo-Fc electron density map (3σ, green meshes) of the C-terminal of SjTGR (PDB: 22FD) subunit A. (C-D) The 2Fo-Fc electron density map (1σ, blue meshes) and the Fo-Fc electron density map (3σ, green meshes) of the C-terminal of SjTGR (PDB: 22FD) subunit B. (TIF) [file ppat.1014125.s006.tif]

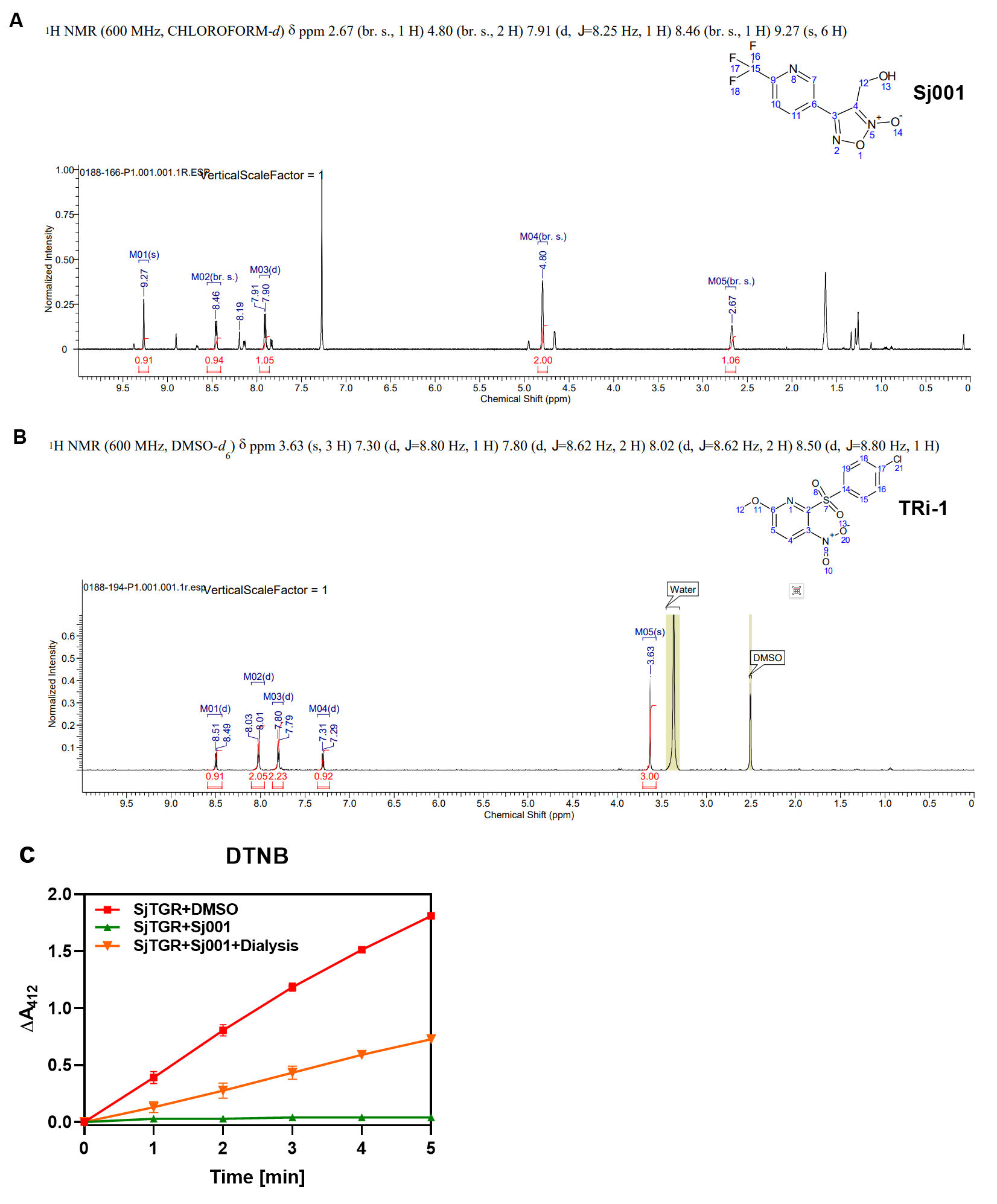

Supplement: S7 Fig — (A) 1H NMR Spectrum of compound Sj001. (B) 1H NMR Spectrum of compound TRi-1. (C) Detection diagram of the reversible inhibition of SjTGR by Sj001 (mean ± SD, n = 3; In some cases, error bars are smaller than the symbol and thus not visible). (TIF) [file ppat.1014125.s007.tif]

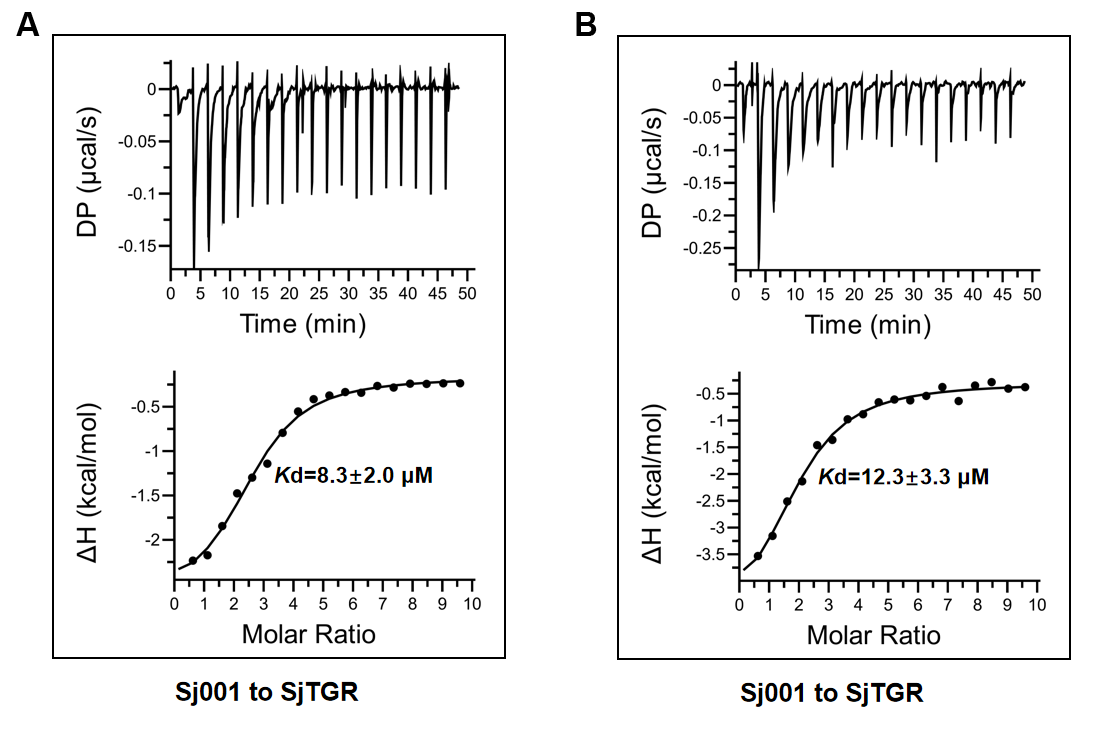

Supplement: S8 Fig — The two additional technical replicates for ITC analysis of Sj001 binding to wild-type SjTGR. (TIF) [file ppat.1014125.s008.tif]

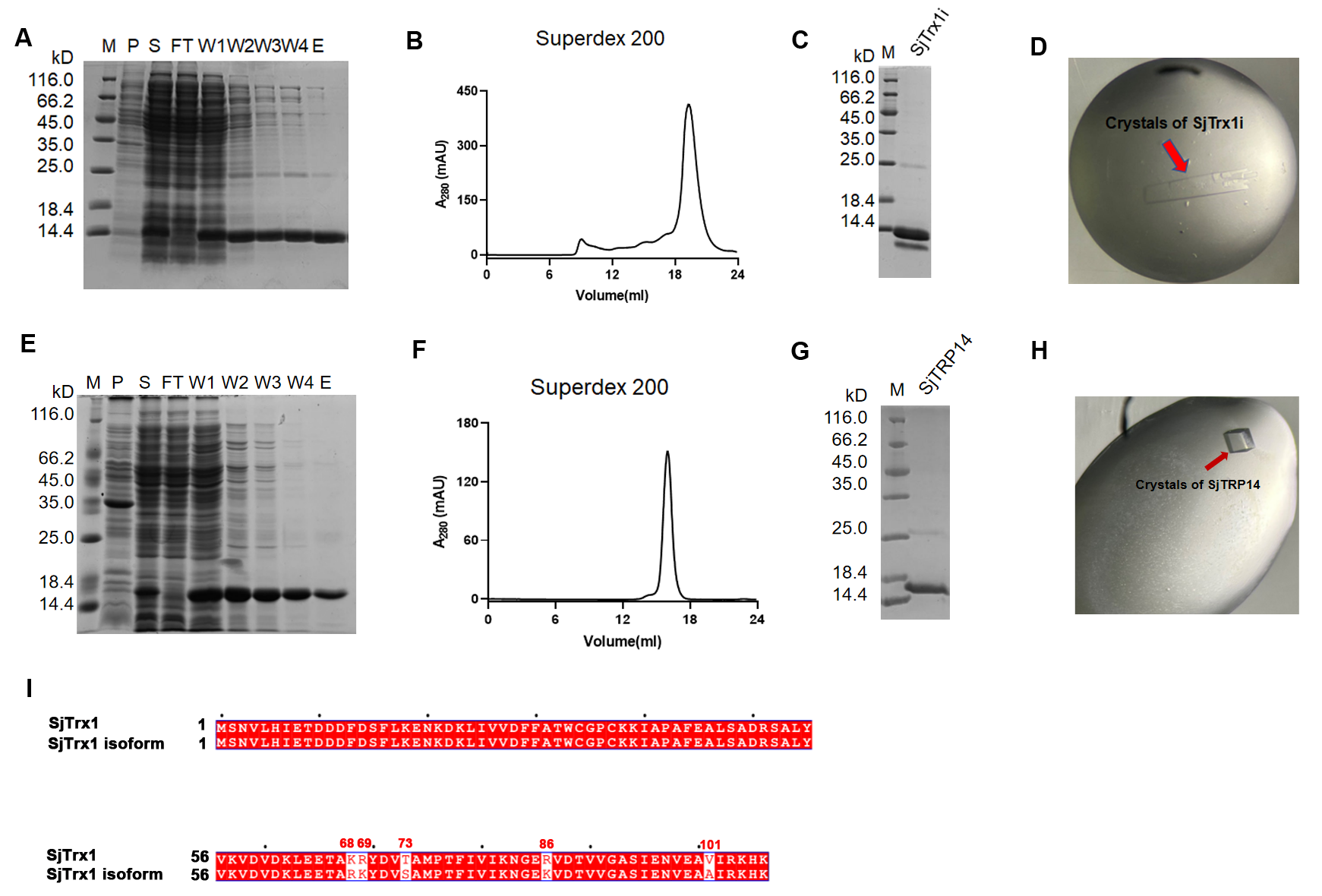

Supplement: S9 Fig — (A) 15% SDS-PAGE analysis of SjTrx1i purification by the Ni-NTA affinity chromatography. (M: marker, P: precipitate, S: supernatant, FT: flow-through, W1–4: wash fractions 1–4, E: eluate). (B) Elution profile of SjTrx1i from Superdex 200 Increase 10/300 GL size-exclusion chromatography. (C) Gel filtration chromatography displaying the purified SjTrx1i protein obtained from both Ni beads affinity chromatography and Superdex 200 size-exclusion filtration. (D) Crystals of the SjTrx1i protein. (E) 15% SDS-PAGE analysis of SjTRP14 purification by the Ni-NTA affinity chromatography. (M: marker, P: precipitate, S: supernatant, FT: flow-through, W1–4: wash fractions 1–4, E: eluate). (F) Elution profile of SjTRP14 from Superdex 200 Increase 10/300 GL size-exclusion chromatography. (G) Gel filtration chromatography showing the purified SjTRP14 protein obtained from Ni beads affinity chromatography and Superdex 200 size-exclusion filtration. (H) Crystals of the SjTRP14 protein. (I) The SjTrx1i gene utilized in this study is an isoform of the previously reported SjTrx1, differing by five amino acid substitutions: K68R, R69K, T73S, R86K, and V101A. (TIF) [file ppat.1014125.s009.tif]

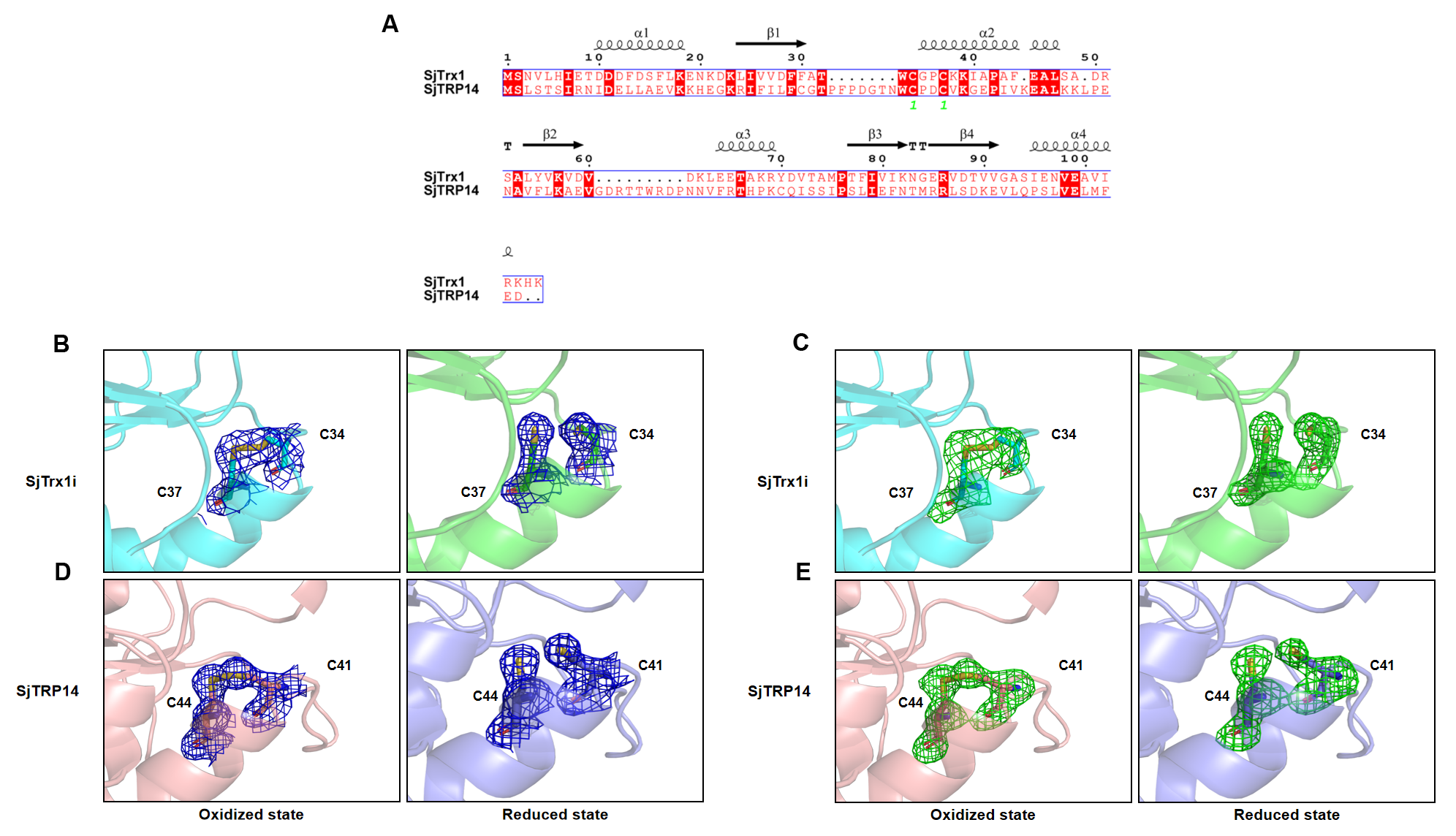

Supplement: S10 Fig — (A) Sequence alignment of SjTrx1i and SjTRP14. (B-C) The 2Fo-Fc electron density maps (1σ, blue meshes) and Fo-Fc electron density maps (3σ, green meshes) for C34 and C37 in the oxidized (PDB: 22FJ) and reduced states (PDB: 22FK) of SjTrx1i are shown. (D-E) The 2Fo-Fc electron density maps (1σ, blue meshes) and Fo-Fc electron density maps (3σ, green meshes) for C41 and C44 in the oxidized (PDB: 22FG) and reduced states (PDB: 22FH) of SjTRP14 are presented. (TIF) [file ppat.1014125.s010.tif]

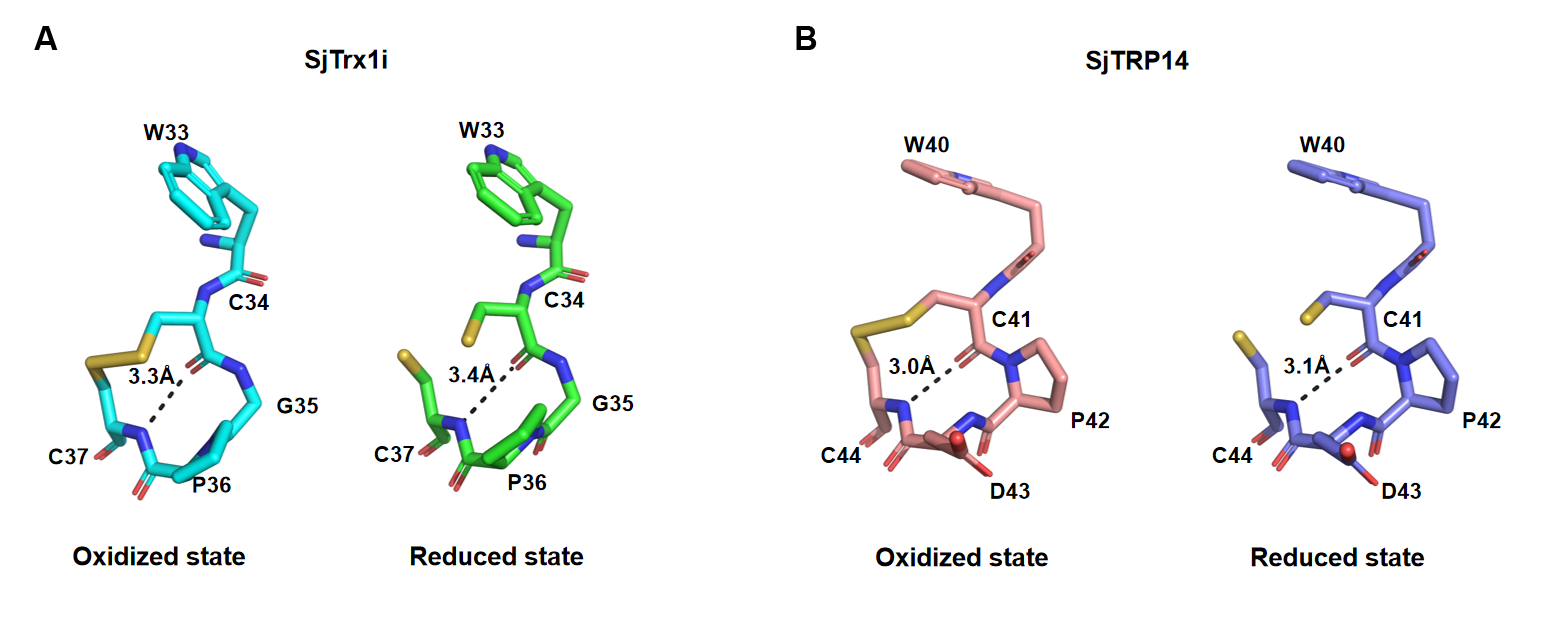

Supplement: S11 Fig — The active sites of the SjTrx1i (A) and SjTRP14 (B) are presented for both their oxidized and reduced states. Distance measurements are indicated by black dashed lines. (TIF) [file ppat.1014125.s011.tif]

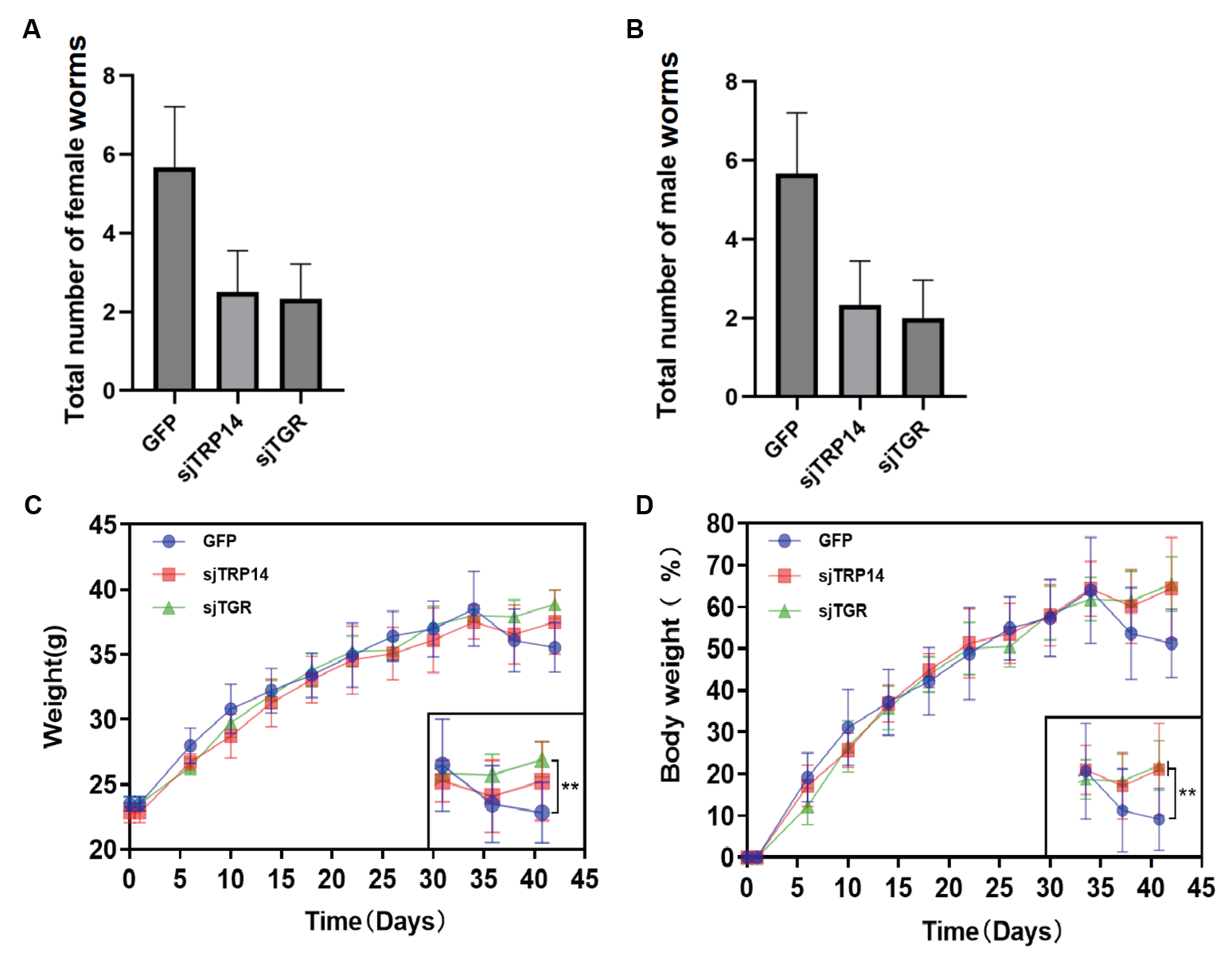

Supplement: S12 Fig — (A-B) The number of female or male worms recovered at 42 dpi in GFP (RNAi), SjTRP14 (RNAi), and SjTGR (RNAi) groups. (C-D) Changes in relative body weight and percentage of weight gain among the three groups. For all the mouse experiments, each group contained six biological replicates, and the data are presented as mean ± SD. (TIF) [file ppat.1014125.s012.tif]
