# Supplementary material for: Structural basis for substrate recognition and inhibition of thioredoxin glutathione reductase from Schistosoma japonicum: Implications for antiparasitic development
Source: PLoS Pathog. 2026 Apr 24;22(4):e1014125. doi: 10.1371/journal.ppat.1014125 (PMC13138743; doi:10.1371/journal.ppat.1014125)
Supplement: S1 Table — (DOCX) [file ppat.1014125.s013.docx]

**S1 Table. Overview of TGR Structures currently available in the PDB.**

| **Protein name** | **Species** | **PDB ID** | **Reference and main conclusion** |
| --- | --- | --- | --- |
| SmTGR | *Schistosoma mansoni* | 2V6O | SmTGR is comprised of two homo-subunits and each subunit is a structural conjugate of a glutaredoxin (Grx) domain and a TrxR [25]. |
| SmTGR | *Schistosoma mansoni* | 2X99 | After binding to TGR, NADPH donates electrons from its nicotinamide ring to FAD interacting with TGR with its isoalloxazine ring and the nearby C154/C159 redox couple for transferring the electrons first to the C596/U597 redox couple on the C-terminal segment of the neighboring subunit, and then to the oxidized Trx or the C28/C31 redox couple of the neighboring monomer to reduce GSSG [26]. |
| SjTGR | *Schistosoma japonicum* | 4LA1 | Only the structure of SjTGR lacking the C-terminal redox center has been determined, and no relevant research articles have been published to date. Current literature is limited to reports concerning its protein purification, crystallization, and diffraction data [28]. |
| EgTGR | *Echinococcus granulosus* | 5W1J | The structural study revealed that TGR functions are achieved not only through a mobile Sec-containing redox center but also by rotation of the Grx domain and distinct binding sites for Grx domain and thioredoxin [43]. |
