# Supplementary material for: Structural basis for substrate recognition and inhibition of thioredoxin glutathione reductase from Schistosoma japonicum: Implications for antiparasitic development
Source: PLoS Pathog. 2026 Apr 24;22(4):e1014125. doi: 10.1371/journal.ppat.1014125 (PMC13138743; doi:10.1371/journal.ppat.1014125)
Supplement: S2 Table — (DOCX) [file ppat.1014125.s014.docx]

**S2 Table. List of primers used in this study.**

| **Primer name** | **Forward / reverse primer (**5'- 3') |
| --- | --- |
| SjTGR-WT-F | AGGTAACATATGCATCATCATCATCATCACATGCCTCCGATT |
| SjTGR-WT-R | CGAATTCCATGGGGTGCAGACCTGCAACCGATTATTAACCCTAGCAACCGGT |
| SjTGR-ΔN-F | AGGTAACATATGCATCATCATCATCATCACTATGACTATGATCTGATA |
| SjTGR-ΔN-R | CGAATTCCATGGGGTGCAGACCTGCAACCGATTATTAACCCTAGCAACCGGT |
| SjTGR-C28S-F | AGCAAAACAACTTCTCCATTTTGCAAA |
| SjTGR-C28S-R | TTTGCAAAATGGAGAAGTTGTTTTGCT |
| SjTGR-R317A-F | ACTGTTATGGTTGCCTCCATTTTGCTT |
| SjTGR-R317A-R | AAGCAAAATGGAGGCAACCATAACAGT |
| SjTGR-U597C-F | GCGCGGCAGCCATATGCCTCCGATTGATGGA |
| SjTGR-U597C-R | TGGTGGTGCTCGAGTTAACCGCAGCAACCGGTTAC |
| SjTGR-T594V-F | TCTGCAGCGGTAGTTGGTTGCTAGGGT |
| SjTGR-T594V-R | ACCCTAGCAACCAACTACCGCTGCAGA |
| SjTGR-U597C/C596S-F | GCGCGGCAGCCATATGCCTCCGATTGATGGA |
| SjTGR-U597C/C596S-R | TGGTGGTGCTCGAGTTAACCGCAGGAACCG |
| SjTrx1i-F | GCGCGGCAGCCATATGAGTAACGTACTGCAT |
| SjTrx1i-R | GTGGTGGTGCTCGAGTCATTTGTGTTTCCG |
| SjTrx1i-C34S-F | TTCGCAACTTGGTCTGGGCCGTGTAAAAAA |
| SjTrx1i-C34S-R | TTTTTTACACGGCCCAGACCAAGTTGCGAA |
| SjTrx1i-C37S-F | TGGTGTGGGCCGTCTAAAAAAATAGCTCCT |
| SjTrx1i-C37S-R | AGGAGCTATTTTTTTAGACGGCCCACACCA |
| SjTRP14-F | AGGAGATATACATATGAGTCTATCTACATCT |
| SjTRP14-R | GTGGTGGTGCTCGAGGTCCTCGAACATGAG |
| SjTRP14-C41S-F | GGGACTAACTGGTCTCCCGATTGCGTTAAA |
| SjTRP14-C41S-R | TTTAACGCAATCGGGAGACCAGTTAGTCCC |
| SjTRP14-C44S-F | TGGTGTCCCGATTCCGTTAAAGGTGAGCCA |
| SjTRP14-C44S-R | TGGCTCACCTTTAACGGAATCGGGACACCA |
| SjTRP14 dsRNA-F | TAATACGACTCACTATAGGGTATCCTCTTCTGTGGTACACC |
| SjTRP14 dsRNA-R | TAATACGACTCACTATAGGGCGATCAGTGATGGAATGCTA |
| SjTGR dsRNA-F | TAATACGACTCACTATAGGGACCTGGAGCAATCGAATATG |
| SjTGR dsRNA-R | TAATACGACTCACTATAGGGCGATAATTGTGGTTCACGAC |
| GFP dsRNA-F | TAATACGACTCACTATAGGGAGAGCAGCACGACTTCTTCAA |
| GFP dsRNA-R | TAATACGACTCACTATAGGGAGAGAACTCCAGCAGGACCAT |
| SjTRP14-qPCR-F | TGAAAGCTGAAGTTGGAGATAG |
| SjTRP14-qPCR-R | GAACATGAGCTCGACAAGACT |
| SjTGR-qPCR-F | CTGGATTATGTAGAACCGACTC |
| SjTGR-qPCR-R | CTGATTTATCCAAGCTCCAAC |
| α-tublin-qPCR-F | CTGATTTTCCATTCGTTTG |
| α-tublin-qPCR-R | GTTGTCTACCATGAAGGC |
