# Supplementary material for: Structural basis for substrate recognition and inhibition of thioredoxin glutathione reductase from Schistosoma japonicum: Implications for antiparasitic development
Source: PLoS Pathog. 2026 Apr 24;22(4):e1014125. doi: 10.1371/journal.ppat.1014125 (PMC13138743; doi:10.1371/journal.ppat.1014125)
Supplement: S3 Table — (DOCX) [file ppat.1014125.s015.docx]

**S3 Table. Crystallographic data collection and refinement statistics.**

|  | SjTGR^WT^ (*EH_4_*) | SjTGR^WT^ (*EH_2_’*) | SjTGR^U597C^ (*EH_2_*) | SjTGR^U597C^ (*E_ox_*) |
| --- | --- | --- | --- | --- |
| **PDB ID** | 9LWM | 22EY | 22FC | 9LWZ |
| **Data collection** |  |  |  |  |
| Wavelength (Å) | 0.91908 | 0.97946 | 0.97918 | 0.97918 |
| Space group | P2_1_2_1_2_1_ | P2_1_2_1_2_1_ | P2_1_2_1_2_1_ | P2_1_2_1_2_1_ |
| Cell dimensions |  |  |  |  |
| *a, b, c* (Å) | 83.62, 86.27, 182.09 | 85.44, 88.16, 185.52 | 84.38, 86.83, 184.47 | 86.81, 86.62, 182.58 |
| *α, β, γ* (°) | 90.00, 90.00, 90.00 | 90.00, 90.00, 90.00 | 90.00, 90.00, 90.00 | 90.00, 90.00, 90.00 |
| Resolution (Å) | 91.05-2.56(2.69-2.56) | 50.63-2.03(2.15-2.03) | 50.65-2.16(2.22-2.16) | 91.29-2.07(2.19-2.07) |
| *R*_merge_ | 0.279(2.602) | 0.134(1.632) | 0.122(1.600) | 0.145(2.136) |
| I/σ (I) | 6.6(1.1) | 14.3(1.5) | 14.5(1.9) | 11.4(1.1) |
| CC½ | 0.988(0.366) | 0.999(0.472) | 0.999(0.649) | 0.998(0.454) |
| No. reflections | 360671 | 1182871 | 947481 | 948745 |
| Completeness (%) | 99.9(99.6) | 99.9(99.5) | 100.0(100.0) | 100.0(100.0) |
| Redundancy | 8.3(6.6) | 12.9(12.6) | 12.9(13.2) | 11.7(9.5) |
|  |  |  |  |  |
| **Refinement** |  |  |  |  |
| *R*_work_/*R*_free_ | 0.2069/0.2599 | 0.2012/0.2459 | 0.1912/0.2294 | 0.1928/0.2389 |
| No. atoms |  |  |  |  |
| Protein | 9095 | 9073 | 9074 | 9065 |
| Ligand/ion | 106 | 107 | 106 | 112 |
| Water | 14 | 333 | 131 | 306 |
| *B*-factors(Å²) |  |  |  |  |
| Protein | 51.499 | 47.024 | 49.373 | 44.936 |
| Ligand/ion | 44.090 | 38.275 | 50.269 | 45.441 |
| Water | 31.549 | 43.707 | 41.786 | 41.800 |
| R.m.s. deviations |  |  |  |  |
| Bond lengths (Å) | 0.0069 | 0.0068 | 0.0065 | 0.0081 |
| Bond angles (°) | 1.6575 | 1.5548 | 1.5474 | 1.6977 |
