# Supplementary material for: Structural basis for substrate recognition and inhibition of thioredoxin glutathione reductase from Schistosoma japonicum: Implications for antiparasitic development
Source: PLoS Pathog. 2026 Apr 24;22(4):e1014125. doi: 10.1371/journal.ppat.1014125 (PMC13138743; doi:10.1371/journal.ppat.1014125)
Supplement: S4 Table — (DOCX) [file ppat.1014125.s016.docx]

**S4 Table. Crystallographic data collection and refinement statistics.**

|  | SjTGR-NADPH | SjTGR-GSH | SjTGR-Au |
| --- | --- | --- | --- |
| **PDB ID** | 22FD | 22FE | 22FF |
| **Data collection** |  |  |  |
| Wavelength (Å) | 0.97923 | 0.97918 | 0.97918 |
| Space group | P2_1_2_1_2_1_ | P2_1_2_1_2_1_ | P2_1_2_1_2_1_ |
| Cell dimensions |  |  |  |
| *a, b, c* (Å) | 84.22, 86.36, 183.59 | 84.56, 87.58, 185.60 86.36, 183.59 | 84.14, 86.55, 184.14 |
| *α, β, γ* (°) | 90.00, 90.00, 90.00 | 90.00, 90.00, 90.00 | 90.00, 90.00, 90.00 |
| Resolution (Å) | 61.20-1.90(1.95-1.90)  2.56) | 50.88-2.12(2.18-2.12) | 50.51-1.84(1.89-1.84) |
| *R*_merge_ | 0.118(1.443) | 0.136(1.957) | 0.096(1.771) |
| I/σ (I) | 16.6(1.8) | 14.0(1.6) | 15.7(1.7) |
| CC½ | 0.999(0.727) | 0.998(0.608) | 0.999(0.645) |
| No. reflections | 1355259 | 1011026 | 1512124 |
| Completeness (%) | 100.0(100.0) | 100.0(100.0) | 100.0(100.0) |
| Redundancy | 12.8(10.6) | 12.8(13.3) | 12.9(13.4) |
|  |  |  |  |
| **Refinement** |  |  |  |
| *R*_work_/*R*_free_ | 0.1898/0.2273 | 0.2089/0.2479 | 0.1902/0.2243 |
| No. atoms |  |  |  |
| Protein | 9119 | 9074 | 9076 |
| Ligand/ion | 182 | 135 | 112 |
| Water | 539 | 132 | 416 |
| *B*-factors(Å²) |  |  |  |
| Protein | 34.539 | 56.733 | 40.818 |
| Ligand/ion | 50.074 | 59.726 | 37.336 |
| Water | 35.237 | 42.739 | 38.857 |
| R.m.s. deviations |  |  |  |
| Bond lengths (Å) | 0.0074 | 0.0087 | 0.0082 |
| Bond angles (°) | 1.5352 | 1.8012 | 1.5871 |
