# Supplementary material for: Structural basis for substrate recognition and inhibition of thioredoxin glutathione reductase from Schistosoma japonicum: Implications for antiparasitic development
Source: PLoS Pathog. 2026 Apr 24;22(4):e1014125. doi: 10.1371/journal.ppat.1014125 (PMC13138743; doi:10.1371/journal.ppat.1014125)
Supplement: S5 Table — (DOCX) [file ppat.1014125.s017.docx]

**S5 Table. Anomalous scattering signals of Au.**

| **Order** | **Height** | **Orthogonal** **coordinates** | **Sequence ID** | **Redox center** |
| --- | --- | --- | --- | --- |
| 1 | 57.45 | 0.88, 62.19,18.27 | 600Au(a) | C28(a)-C31(a) |
| 2 | 27.99 | 51.70, 57.68, 44.36 | 601Au(a) | C596(a)-U/C597(a) |
| 3 | 24.58 | 41.69, 83.62, 24.12 | 600Au(b) | C28(b)-C31(b) |
| 4 | 14.37 | 30.68, 31.96, 11.82 | 602Au(a) | C154(a)-C159(a) |
| 5 | 14.19 | 18.20, 34.76, 6.66 | 601Au(b) | C596(b)-U/C597(b) |
| 6 | 12.30 | 20.59, 13.47, 42.23 | 602Au(b) | C154(b)-C159(b) |
| 7 | 11.31 | 20.99, 35.00, 8.29 | 601Au(b) | C596(b)-U/C597(b) |
