# Supplementary material for: Structural basis for substrate recognition and inhibition of thioredoxin glutathione reductase from Schistosoma japonicum: Implications for antiparasitic development
Source: PLoS Pathog. 2026 Apr 24;22(4):e1014125. doi: 10.1371/journal.ppat.1014125 (PMC13138743; doi:10.1371/journal.ppat.1014125)
Supplement: S6 Table — (DOCX) [file ppat.1014125.s018.docx]

**S6 Table. Crystallographic data collection and refinement statistics.**

|  | SjTrx1i-oxidized | SjTrx1i-reduced | SjTRP14- oxidized | SjTRP14-reduced |
| --- | --- | --- | --- | --- |
| **PDB ID** | 22FJ | 22FK | 22FG | 22FH |
| **Data collection** |  |  |  |  |
| Wavelength (Å) | 0.97853 | 0.97918 | 0.97853 | 0.97853 |
| Space group | P2_1_2_1_2_1_ | P2_1_2_1_2_1_ | P2_1_2_1_2 | P2 |
| Cell dimensions |  |  |  |  |
| *a, b, c* (Å) | 39.53, 90.53, 150.04 | 39.92, 90.86, 151.37 | 52.63, 54.83, 37.75 | 53.44, 38.62, 56.15 |
| *α, β, γ* (°) | 90.00, 90.00, 90.00 | 90.00, 90.00, 90.00 | 90.00, 90.00, 90.00 | 90.00, 90.04, 90.00 |
| Resolution (Å) | 43.38-2.66(2.71-2.66)  2.56) | 45.43-1.90(2.01-1.90) | 37.97-1.69(1.73-1.69) | 38.72-1.77(1.82-1.77) |
| *R*_merge_ | 0.147(0.601) | 0.128(1.961) | 0.073 (0.850) | 0.094 (0.900) |
| I/σ (I) | 7.6(2.2) | 11.9 (1.0) | 15.3 (1.9) | 7.9 (1.4) |
| CC½ | 0.970(0.667) | 0.999(0.472) | 0.999 (0.484) | 0.995 (0.482) |
| No. reflections | 59728 | 415662 | 146434 | 136985 |
| Completeness (%) | 95.7(92.0) | 96.7(97.8) | 100 (99.6) | 98.3 (95.3) |
| Redundancy | 3.8(3.7) | 9.6(9.2) | 11.5 (8.7) | 6.2 (5.3) |
|  |  |  |  |  |
| **Refinement** |  |  |  |  |
| *R*_work_/*R*_free_ | 0.2211/0.2845 | 0.2211/0.2538 | 0.2017/0.2423 | 0.2116/0.2763 |
| No. atoms |  |  |  |  |
| Protein | 4161 | 4169 | 974 | 1938 |
| Ligand/ion | 0 | 0 | 0 | 0 |
| Water | 49 | 120 | 48 | 57 |
| *B*-factors(Å²) |  |  |  |  |
| Protein | 52.033 | 48.156 | 30.287 | 36.738 |
| Ligand/ion | - | - | - | - |
| Water | 25.339 | 38.883 | 33.349 | 37.084 |
| R.m.s. deviations |  |  |  |  |
| Bond lengths (Å) | 0.0072 | 0.0068 | 0.0085 | 0.0068 |
| Bond angles (°) | 1.5386 | 1.5675 | 1.7293 | 1.6160 |
