# Supplementary material for: Structural basis for substrate recognition and inhibition of thioredoxin glutathione reductase from Schistosoma japonicum: Implications for antiparasitic development
Source: PLoS Pathog. 2026 Apr 24;22(4):e1014125. doi: 10.1371/journal.ppat.1014125 (PMC13138743; doi:10.1371/journal.ppat.1014125)
Supplement: S7 Table — (DOCX) [file ppat.1014125.s019.docx]

**S7 Table. Quantification of Selenium incorporation efficiency by ICP-MS.**

| **Replicate** | **Measured Se (μg/L)** | **Se Molar Conc. (nM)** | **Protein Molar Conc. (μM)** | **Incorporation Rate (%)** |
| --- | --- | --- | --- | --- |
| 1 | 72.62 | 919.76 | 1.00 | 91.98 |
| 2 | 69.89 | 885.15 | 1.00 | 88.52 |
| 3 | 76.13 | 964.13 | 1.00 | 96.41 |
