# Supplementary material for: Structural basis for substrate recognition and inhibition of thioredoxin glutathione reductase from Schistosoma japonicum: Implications for antiparasitic development
Source: PLoS Pathog. 2026 Apr 24;22(4):e1014125. doi: 10.1371/journal.ppat.1014125 (PMC13138743; doi:10.1371/journal.ppat.1014125)
Supplement: S1 File — (DOCX) [file ppat.1014125.s020.docx]

**SjTGR sequence:**

ATGCCTCCGATTGATGGAACATCCCAGTGGTTGCAGAGGACTATCGAATCAGCGGCGGTAATCGTCTTTAGCAAAACAACTTGTCCATTTTGCAAAAAGCTAAAGGATGTTTTAGCTGAAGCAAAGATTAAACACGCTACAATTGAACTGGATCAGTTATCCAATGGTTCGGTTATTCAAAAGGCCTTATCTAGCTTTTCTAAAATTGAAACAGTCCCGCAAATGTTTGTTAGAGGCAAGTTCATTGGCGATTCTAAAGCAGTACTTAATTACCACAATAATAATCAATTGCAGGCGATCGTCAACGAAAATAAGTATGACTATGATCTGATAATCATCGGTGGAGGATCTGGTGGACTCGCTGCTGGAAAGGAGGCAGCCAAATACGGCGCAAAGACAGCTGTTCTGGATTATGTAGAACCGACTCCAATGGGTACTACTTGGGGATTAGGTGGAACCTGTGTTAACGTTGGATGTATCCCTAAAAAATTAATGCACCAAGCTGGACTCTTAAGTCATTCTTTGGAAGATGCCCAACATTTCGGTTGGAGCTTGGATAAATCAAAAATTTCCCATGATTGGTCAACTATGGTTGAAGGAGTTCAGAGTCACATCGGTTCTTTAAATTGGGGCTATAAAGTTTCACTAAGAGATAATGCGGTTACATATCTTAATGCTCGTGGGATGCTATTAAGTTCTCATGAGGTTCAGATTACAGAAAAGAATAAAAAAGTATCCACAATAACTGGAAATAAAATCATCTTAGCTACTGGCGAGCGTCCAAAATACCCAGAAATACCTGGAGCAATCGAATATGGGATTACAAGTGATGATTTGTTTTCCTTACCATACTTCCCGGGCAAAACACTGGTCGTTGGAGCGAGCTATGTTGCATTGGAATGTGCTGGTTTTCTTGCCAGTTTGGGCGGTGATGTTACTGTTATGGTTCGTTCCATTTTGCTTCGTGGTTTCGATCAACAAATGGCTGAGAAGGTTGGCGACTATATGGAAAATCATGGAGTCAAGTTCGCAAAGTTGTGTGTACCAGACGAGATTACACAGTTGAAACCGGTAGATACTGAGAATAACAAACCTGGACTCCTGCTTGTTAAGGGTCATTATACTGATGGTAAGAAGTTTGAAGAAGAATTTGAAACGGTCATTTTCGCTGTTGGTCGTGAACCACAATTATCGAAGCTTAATTGTGAAGCTGTCGGTGTTAAACTAGATAAGAATGGTCGGGTTGTATGCTCAGATGATGAACAAACTACAGTCAGTAACATTTATGCCATTGGAGATATAAACGCTGGAAAACCACAGTTAACTCCAGTGGCTATTCATGCTGGACGTTATTTGGCTAGACGGTTATTCGCTGGTGCAACTGAACTGACTGACTATTCCAATGTTGCTACGACTGTTTTCACTCCATTAGAATATGGCGCTTGTGGACTGAGTGAAGAGGATGCAATTGAAAAGTATGGTGATAATGATATCGAGGTATATCATTCACATTTCAAACCTTTAGAATGGACTGTTGCTCATCGTGAAGATAATGTTTGTTACATGAAACTTGTTTGCCGTATATCTGATAACATGCGTGTACTGGGTCTACATGTTTTAGGACCTAATGCAGGTGAAATAACACAGGGGTATGCAGTTGCAATTAAAATGGGTGCAACTAAAGAAGATTTTGATCGTACCATAGGAATTCACCCAACTTGTTCTGAGACATTTACAACGTTGCATGTAACCAAGAGATCTGGGGGCTCTGCAGCGGTAACCGGTTGCTAGGGTTAA

MPPIDGTSQWLQRTIESAAVIVFSKTTCPFCKKLKDVLAEAKIKHATIELDQLSNGSVIQKALSSFSKIETVPQMFVRGKFIGDSKAVLNYHNNNQLQAIVNENKYDYDLIIIGGGSGGLAAGKEAAKYGAKTAVLDYVEPTPMGTTWGLGGTCVNVGCIPKKLMHQAGLLSHSLEDAQHFGWSLDKSKISHDWSTMVEGVQSHIGSLNWGYKVSLRDNAVTYLNARGMLLSSHEVQITEKNKKVSTITGNKIILATGERPKYPEIPGAIEYGITSDDLFSLPYFPGKTLVVGASYVALECAGFLASLGGDVTVMVRSILLRGFDQQMAEKVGDYMENHGVKFAKLCVPDEITQLKPVDTENNKPGLLLVKGHYTDGKKFEEEFETVIFAVGREPQLSKLNCEAVGVKLDKNGRVVCSDDEQTTVSNIYAIGDINAGKPQLTPVAIHAGRYLARRLFAGATELTDYSNVATTVFTPLEYGACGLSEEDAIEKYGDNDIEVYHSHFKPLEWTVAHREDNVCYMKLVCRISDNMRVLGLHVLGPNAGEITQGYAVAIKMGATKEDFDRTIGIHPTCSETFTTLHVTKRSGGSAAVTGCUG

**SjTrx1i sequence:**

ATGAGTAACGTACTGCATATAGAAACCGATGACGATTTTGATTCTTTCTTAAAGGAAAATAAGGATAAATTAATTGTTGTTGATTTTTTCGCAACTTGGTGTGGGCCGTGTAAAAAAATAGCTCCTGCTTTCGAAGCGTTGAGCGCTGATCGTTCGGCATTATATGTGAAGGTTGACGTGGATAAACTTGAAGAAACTGCCAAAAGATACGATGTAACAGCTATGCCAACGTTTATTGTGATAAAAAATGGCGAAAGAGTCGATACAGTGGTTGGAGCTTCTATAGAAAATGTTGAAGCTGTGATCCGGAAACACAAATGA

MSNVLHIETDDDFDSFLKENKDKLIVVDFFATWCGPCKKIAPAFEALSADRSALYVKVDVDKLEETAKRYDVTAMPTFIVIKNGERVDTVVGASIENVEAVIRKHK

**SjTRP14 sequence:**

ATGAGTCTATCTACATCTATCAGAAATATCGACGAACTTTTGGCTGAAGTGAAAAAACACGAAGGTAAAAGGATATTTATCCTCTTCTGTGGTACACCTTTCCCAGATGGGACTAACTGGTGTCCCGATTGCGTTAAAGGTGAGCCAATAGTGAAAGAAGCACTTAAAAAATTGCCAGAAAATGCAGTCTTTTTGAAAGCTGAAGTTGGAGATAGAACCACTTGGCGTGATCCGAACAATGTGTTTAGAACACATCCAAAATGTCAAATAAGTAGCATTCCATCACTGATCGAGTTTAATACTATGAGAAGATTGTCAGACAAGGAAGTTTTGCAACCTAGTCTTGTCGAGCTCATGTTCGAGGACTAG

MSLSTSIRNIDELLAEVKKHEGKRIFILFCGTPFPDGTNWCPDCVKGEPIVKEALKKLPENAVFLKAEVGDRTTWRDPNNVFRTHPKCQISSIPSLIEFNTMRRLSDKEVLQPSLVELMFED
