# Supplementary material for: Structural basis for substrate recognition and inhibition of thioredoxin glutathione reductase from Schistosoma japonicum: Implications for antiparasitic development
Source: PLoS Pathog. 2026 Apr 24;22(4):e1014125. doi: 10.1371/journal.ppat.1014125 (PMC13138743; doi:10.1371/journal.ppat.1014125)
Supplement: Attachment — Submitted filename: Response to reviewers comments.docx [file ppat.1014125.s022.docx]

Additional Editor Comments:

As you will see, all three reviewers acknowledge the new insight this study provides and its potential impact. However, all reviewers raise important concerns regarding both the experimental work and the presentation, which would need to be addressed in detail.

We thank the reviewers for their helpful comments and the editor for handling this manuscript, which have greatly helped us to improve the quality of our manuscript. We have carefully considered each point and have made corresponding revisions to the manuscript. Our point-by-point responses are provided below.

Journal Requirements:

1) Please provide an Author Summary. This should appear in your manuscript between the Abstract (if applicable) and the Introduction, and should be 150-200 words long. The aim should be to make your findings accessible to a wide audience that includes both scientists and non-scientists. Sample summaries can be found on our website under Submission Guidelines:

https://journals.plos.org/plospathogens/s/submission-guidelines#loc-parts-of-a-submission

We apologize for the oversight in omitting this file. An Author Summary has now been added to the revised manuscript. Please see the details below:

Schistosomiasis, a devastating neglected tropical disease (NTD), affects approximately 240 million individuals and puts over 700 million at risk. While praziquantel (PZQ) is the only available therapeutic, it faces significant limitations, highlighting an urgent need to discover novel drug targets and develop new anti-schistosomal agents. Thioredoxin glutathione reductase (TGR), essential for parasite survival, is a promising target; however, structural insights into *S. japonicum* TGR (SjTGR) remain limited, hampering targeted drug development. Here, we report the complete catalytic cycle of SjTGR, resolved by X-ray crystallography, establishing the structural basis of its mechanism. Functional studies confirm its critical role in maintaining schistosome survival and fecundity. By elucidating its unique catalytic architecture including a tripartite active site, T594-mediated electron transfer enhancement, and R317-dependent NADPH stabilization, this work reveals schistosome-specific redox adaptations. These structural insights distinguish SjTGR from its mammalian orthologs and provide a robust foundation for designing selective therapeutics against schistosomiasis, therefore contributing to the global elimination of this disease burden.

2) Please upload all main figures as separate Figure files in .tif or .eps format. For more information about how to convert and format your figure files please see our guidelines:

https://journals.plos.org/plospathogens/s/figures

Thank you for your suggestion. We have uploaded all main figures as separate Figure files in .tif format in our revised submission, ensuring compliance with the journal's figure submission guidelines.

3) We have noticed that you have uploaded Supporting Information files, but you have not included a list of legends. Please add a full list of legends for your Supporting Information files after the references list.

As requested, a full list of legends for all Supporting Information files has been appended after the References section in the revised manuscript.

4) Some material included in your submission may be copyrighted. According to PLOSu2019s copyright policy, authors who use figures or other material (e.g., graphics, clipart, maps) from another author or copyright holder must demonstrate or obtain permission to publish this material under the Creative Commons Attribution 4.0 International (CC BY 4.0) License used by PLOS journals. Please closely review the details of PLOSu2019s copyright requirements here: PLOS Licenses and Copyright. If you need to request permissions from a copyright holder, you may use PLOS's Copyright Content Permission form.

Please respond directly to this email and provide any known details concerning your material's license terms and permissions required for reuse, even if you have not yet obtained copyright permissions or are unsure of your material's copyright compatibility. Once you have responded and addressed all other outstanding technical requirements, you may resubmit your manuscript within Editorial Manager.

Potential Copyright Issues:

- Please confirm (a) that you are the photographer of Figure 6., or (b) provide written permission from the photographer to publish the photo(s) under our CC BY 4.0 license.

ii) Figure Figure 6.. Please confirm whether you drew the images / clip-art within the figure panels by hand. If you did not draw the images, please provide (a) a link to the source of the images or icons and their license / terms of use; or (b) written permission from the copyright holder to publish the images or icons under our CC BY 4.0 license. Alternatively, you may replace the images with open source alternatives. See these open source resources you may use to replace images / clip-art:

- https://commons.wikimedia.org

- https://openclipart.org/.

We thank you for raising this critical issue regarding copyright compliance. In the revised manuscript, we have replaced the images in Figure 6A with an open-source alternative, which are available under appropriate licenses on Wikimedia Commons (https://commons.wikimedia.org/) and Openclipart (https://openclipart.org/). This update fully aligns with the journal's copyright policy. We have also added a source attribution statement to the figure legend for Fig. 6A to ensure full compliance with open-source image usage policies. The legend now includes the following clarification:

"The images are from Wikimedia Commons (https://commons.wikimedia.org/) and Openclipart (https://openclipart.org/)."

5) Please amend your detailed Financial Disclosure statement. This is published with the article. It must therefore be completed in full sentences and contain the exact wording you wish to be published.

State what role the funders took in the study. If the funders had no role in your study, please state: "The funders had no role in study design, data collection and analysis, decision to publish, or preparation of the manuscript.".

If you did not receive any funding for this study, please simply state: u201cThe authors received no specific funding for this work.u201d

Thank you for highlighting this point. In accordance with the journal's policy, we have added the following statement to the revised manuscript (in the Funding section): “The funders had no role in study design, data collection and analysis, decision to publish, or preparation of the manuscript.”.

Reviewers' Comments:

Reviewer's Responses to Questions

Part I - Summary

Please use this section to discuss strengths/weaknesses of study, novelty/significance, general execution and scholarship.

Reviewer #1: This study resolves the structures to *Schistosoma japonicum* thioredoxin glutathione reductase (SjTGR) in combination with various cofactors, substrates, and the anti-schistosomal compound auranofin. The crystal data provide resolution into enzymatic mechanism and new insight into the mechanism of action of auranofin. While structural insight into TGR is already available from other schistosome species (PDB:7B02), the breadth and resolution of this work provides new detail and is a useful comparison for the *S. mansoni* complex.

Thank you very much for the positive comments.

Reviewer #2: This manuscript, “Structural Basis for Substrate Recognition and Inhibition of Thioredoxin Glutathione Reductase from Schistosoma japonicum”, provides a comprehensive structural and biochemical analysis of SjTGR, a parasite-specific selenoenzyme essential for redox homeostasis. SjTGR as a drug target for schistosomiasis. The authors present eleven crystal structures of SjTGR in multiple redox states and in complex with NADPH, GSH, and Auranofin, supported by mutagenesis, enzymatic assays, and in vivo RNAi experiments. They show that a unique hook-shaped C-terminal motif stabilized by residue T594 of SjTGR fine-tunes electron transfer. R317 is shown to be critical for NADPH binding, while C28 in the Grx domain is indispensable for GSH-dependent activity. Structural data reveal that the inhibitor Auranofin binds simultaneously to three redox centers, explaining its action. Activity assays of SjTGR’s substrates SjTrx1i and SjTRP14 highlight distinct recognition modes, and RNAi knockdown of SjTGR or SjTRP14 impairs parasite survival, reducing pathology in mice. This is substantial work providing new insight into SjTGR function. However, the analysis of the structural data requires clarification.

We sincerely thank the reviewer for the positive feedback and constructive suggestions. All comments have been carefully considered. In particular, we have substantially revised the manuscript to clarify the analysis of the structural data as recommended.

Reviewer #3: The authors undertake a thorough structural/functional analysis of potential drug targets for Schistosoma japonicum. The study is overall well presented and designed and for the most part, fairly clearly presented. I congratulate the authors on their interesting work. I do have a variety of minor comments and suggestions below.

We sincerely appreciate your encouraging feedback and have carefully addressed all your comments in the revised manuscript, following your suggestions.

Part II – Major Issues: Key Experiments Required for Acceptance

Please use this section to detail the key new experiments or modifications of existing experiments that should be absolutely required to validate study conclusions.

Generally, there should be no more than 3 such required experiments or major modifications for a "Major Revision" recommendation. If more than 3 experiments are necessary to validate the study conclusions, then you are encouraged to recommend "Reject".

Reviewer #1: Major

While the structural data is well elaborated, a major issue is one of rigor of presentation of the functional data. In many places it is unclear from text and figure legends the number of assays and replicates that have been performed, and as such whether errors are quoted as sd or sem. This needs to be clearly elaborated throughout the manuscript for all substrate assays and especially the animal work, including the in vivo RNAi where methods are poorly described. The numbers of mice and worms (Fig 6B&C), the number of relevant replicates used for qPCR analyses need to be clearly outlined to ensure replicability. Does the sex bias refer to recovered worms or mice (Fig 6B,C)? How many tail injections were performed (10?). If there are 6 mice per group, how many groups are shown? The graph axis in 6D refers to ‘insects’ which is surely incorrect! Are these data normalized to naïve worms or anti-GFP worms?

We are grateful to the reviewer for these critical and helpful comments. Addressing them has substantially enhanced the clarity and rigor of our data presentation.

We acknowledge that the initial manuscript lacked explicit details on replicate numbers and error bar definitions. We have now thoroughly addressed this in the revised version as follows:

(1) Enzymatic/Substrate Assays: All enzymatic and substrate assays were performed in triplicate. Data are presented as the mean ± SD, as now explicitly stated in the respective Methods section and figure legends (e.g., “mean ± SD, n=3”).
(2) Mouse Experiments: For all *in vivo* studies, each experimental group contained six biological replicates. Results are presented as the mean ± SD. This detail has been added to the Methods and figure legends pertaining to animal data.
(3) qPCR Analysis: All qPCR experiments were conducted in triplicate, with data expressed as the mean ± SD. This information has been incorporated into the relevant Methods section and figure legends.

These revisions ensure full transparency regarding experimental replicates and statistical reporting throughout the manuscript.

For the Fig 6B&C, two pairs of adult worms (males and females separated) harvested from six mice in each group were used for qPCR analysis of the RNAi efficacy for SjTGR and SjTRP14. qPCR analysis was performed in three technical replicates using specifically designed primers. Results are presented as the mean ± SD. These methodological details have been explicitly added to the relevant Methods section and figure legends in the revised manuscript.

In response to the comment, we have restructured and expanded the description of the *in vivo* RNAi experiments to provide the necessary methodological detail in the revised manuscript: “Following infection with 30 ± 2 cercariae, 18 infected female Kunming mice were randomly divided into three groups (n=6 per group): the normal control group (GFP dsRNA), sjTRP14 group (sjTRP14 dsRNA), and sjTGR group (sjTGR dsRNA). Subsequently, each mouse was administered 10 μg of dsRNA (dissolved in 200 μL physiological saline) via intravenous tail vein injection on the following days post-infection (dpi): 1, 6, 10, 14, 18, 22, 26, 30, 34, and 38, respectively. This dose and schedule were selected based on preliminary studies [54-55] to effectively inhibit target gene expression in *S. japonicum* for the duration of the treatment period. Samples were collected from all the experimental mice at 42 dpi. Control mice were injected with dsRNA of *gfp*, a green fluorescent protein from *Aequorea victoria* that is not present in *S. japonicum* [55].”

We thank the reviewer for identifying the error in Figure 6D. The y-axis label has been corrected to “Number of adult worms” in the revised figure, which has been replaced in the resubmission. This correction does not affect any of the study's conclusions. We apologize for the oversight.

The GFP dsRNA-treated group was used as a negative control in this study. Accordingly, these experimental data were normalized to the values obtained from GFP-RNAi worms.

Reviewer #2: A major advantage of this study is that they employ the native Sec-containing SjTGR for both structural characterization and enzymatic assays. However, it is key to show that indeed the enzyme used in this study was pure and primarily Sec-containing. While the presence of Sec in position U597 is shown in supporting S1, it does not rule out the minor presence of other forms of SjTGR, with possibility of minor misincorporation of serine and other amino acids at position, or truncation at position 597. The purity of the preparation and the percentage of Sec incorporation would influence the kinetic parameters reported in Table 1. An intact protein mass spectrometry of the batches used in the experiments is essential for supporting the conclusions drawn in the manuscript.

We sincerely thank the reviewer for raising these critical and constructive questions. We agree that it is crucial to demonstrate that the enzyme preparation SjTGR used in this study was highly pure and predominantly contained selenocysteine (Sec). Regarding the expression of recombinant SjTGR^WT^, we adapted the method from Qing et al. (PMID: 28193838; 35101220), which utilizes a genomically recoded *E. coli* strain (C321.ΔA) with a depleted RF1 to incorporate selenocysteine at a predefined UAG codon, enabling high-yield production of selenoproteins (approximately 100% Sec incorporation). As suggested, we have now elaborated on this methodology in the revised Methods section.

The obtained recombinant SjTGR^WT^ protein was purified by Ni-affinity chromatography column, followed by size-exclusion chromatography column with a Superdex 200 Increase 10/300 GL. SDS-PAGE analysis confirmed the high purity of the protein, which was suitable for subsequent structural and functional studies. The results for expression and purification of recombinant SjTGR^WT^ have been incorporated into Fig. S1 in the revised manuscript.

Given the critical role of the selenocysteine (Sec) residue in the C-terminal redox motif for TGR catalysis and electron transfer, we assessed the activity of our recombinant enzyme. As shown in Fig. 1L, the wild-type enzyme SjTGR^WT^ exhibited robust DTNB reductase activity, whereas the Sec-to-Cys mutant (SjTGR^U597C^) was catalytically inactive. This functional contrast provides direct evidence for the successful incorporation of Sec into our purified SjTGR^WT^ protein.

Furthermore, to verify the selenocysteine incorporation essential for enzymatic activity, we quantified the selenium content in our purified SjTGR^WT^ protein batch using inductively coupled plasma mass spectrometry (ICP-MS). The analysis confirmed a selenocysteine incorporation efficiency of 92.3% (the mean of triplicate measurements), validating the protein's suitability for functional assays. We have provided the data in the Table S7 of our revised submission. We have also provided a detailed description of the ICP-MS analysis in the Methods section of the revised manuscript.

**Table S7. Quantification of Selenium incorporation efficiency by ICP-MS.**

| **Replicate** | **Measured Se (μg/L)** | **Se Molar Conc. (nM)** | **Protein Molar Conc. (μM)** | **Incorporation Rate (%)** |
| --- | --- | --- | --- | --- |
| 1 | 72.62 | 919.76 | 1.00 | 91.98 |
| 2 | 69.89 | 885.15 | 1.00 | 88.52 |
| 3 | 76.13 | 964.13 | 1.00 | 96.41 |

The second point is the decision process of deducing the presence or absence of disulfide and selenylsulfide bonds in the redox centers. First the material and methods and table S2 seem to suggest that crystallization was set only under oxidizing conditions without reducing reagents. The text (lines 175-177 states “The structures of SjTGRWT and SjTGRU597C in four redox states were analyzed, which included the fully oxidized state (Eox), two intermediate states reduced with two electrons (EH2), and the fully reduced state with four electrons (EH4).” How were these different redox states EH4, EH2’, EH2 and Eox obtained experimentally? Tables S2, S3 and S5 should either include the PDB IDs and sample conditions or those should be added in a separate panel. The rationale for deciding whether a disulfide or selenylsulfide bond is present or not is not fully explained (distances and electron density?). Mobility could also contribute to missing electron density, so the B factors should be displayed. Sample heterogeneity could also be a factor (a mixture of redox states?). Complicating the ability of the reader to judge the data is the unclear graphics in Figure 1 panels D-G. The electron density should be made more transparent for easier visualization. Avoid the use of yellow for atoms other than sulfur (for example see cofactors) or distances. It would help if the PDB IDs were included in the captions per panel.

All of this makes evaluating the data confusing. While Figure 1 panel I is considered the reduced state of the wild type (C-terminus residues GCUG), to this reviewer it appears to be in the same conformation as the oxidized form of the GCCG variant. Certainly, the CU Figure S3I appears to be in a selenysulfide bond and not reduced. Indeed, the high reactivity of Sec necessitates that the selenylsulfide bond reform rapidly, even if the reduced enzyme was used to set the crystal trays (under conditions reported to be depleted of reducing agents). In 2 days, if crystallization, the selenylsulfide bond CU should be the exclusive form. Since the criteria by which this was decided are not discussed, the reader is left ambiguous whether indeed (lines 186-187) the “C-terminal selenothiol motif (C596-U597/C597) in SjTGR adopted a cis-conformation in both oxidized and reduced states”. It may be because the CU is defined as reduced in the S3 panel I, even though it is possibly oxidized.

We sincerely thank the reviewer for these thorough and constructive comments, which have greatly helped us improve the clarity and rigor of our structural analysis. We address each point in detail below, together with the revisions made to the manuscript.

SjTGR^WT^ and SjTGR^U597C^ were crystallized without any added reducing agent, using conditions consistent with those described in the Methods section: 4% Tacsimate (pH 6.5–8.0) and 15–20% PEG 3350. Only minor variations in Tacsimate pH and PEG 3350 concentration were observed, which we confirmed do not alter the protein redox state; thus, these slight differences are not further detailed. The four distinct redox states (*E_ox_*, *EH_2_*, *EH_2_'*, *EH_4_*) were obtained through intrinsic oxidation during crystallization: the recombinant proteins were initially purified in a reduced state (from the reductive cytoplasm of Escherichia coli), and gradual air oxidation during crystal growth and soaking generated the partially oxidized (*EH_2_*, *EH_2_'*) and fully oxidized (*E_ox_*) states, while a subset of crystals remained stably reduced (*EH_4_*) without re-oxidation. This natural redox progression during crystallization allowed us to capture all four states under identical basic crystallization conditions.

In total, nearly 60 crystal structures of SjTGR-WT and SjTGR-U597C were determined. Model building was performed in Coot, followed by multiple rounds of refinement with REFMAC5 (CCP4 suite), including positional refinement, B-factor refinement, geometric restraint refinement, and occupancy refinement. Key cysteine and selenocysteine residues involved in disulfide/selenylsulfide formation were modeled as discrete, non-bonded atoms during occupancy refinement. This modeling scheme yielded the cleanest 2*Fo–Fc* and *Fo–Fc* electron density maps. Although the electron density remained continuous for these residue pairs, no residual positive density supported the presence of a covalent disulfide or selenylsulfide bond, providing additional validation for our redox state assignments. The assignment of disulfide/selenylsulfide bonds and protein redox states was based on two strict criteria: (1) refined interatomic distances: canonical disulfide/selenylsulfide bonds show S–S/S–Se lengths of 2.02–2.2 Å, whereas reduced cysteine/selenocysteine pairs exhibit distances >2.2 Å; (2) the shape and continuity of electron density for the S–S/S–Se moieties.

Notably, even though no reducing agent was used during purification or crystallization, most crystals remained in a reduced state, supported by two lines of evidence: first, recombinant proteins expressed in *E. coli* are inherently reduced due to the cytoplasmic reductive environment; second, the electron densities of C28 and C31 are fully segregated in both Fig. 1E and Fig. 1G, clearly indicating a reduced state rather than a covalent disulfide bond.

Regarding the continuous electron density of C154–C159 and C596–U/C597 in their reduced states, this observation is consistent with our previous structural data: although the S–S/S–Se separation approaches 3.0 Å (far beyond the range of a covalent bond), the electron density remains connected (see the Figure below). The reduced form of C596–C597 adopts a cis-conformation, consistent with the reduced C596–U597 in this work (Figure. 10A). As further supporting evidence, the electron density of oxidized C596–C597 is smooth and continuous (Figure. S3H), whereas reduced C596–U597 shows a characteristic density profile—slightly concave in the middle and bulged at both ends (Figure. S3I)—distinctly different from a covalent selenylsulfide bond. We emphasize that the cis-conformation is an intrinsic structural feature of the C-terminal motif, shared by both oxidized and reduced states, and should not be misinterpreted as evidence of oxidation.

**Structural characterization of the redox centers in SjTGR.** Representative 2***Fo***-***Fc*** electron density maps (blue mesh, contoured at 1σ) are shown for (A) the C-terminal redox center (C596–C597) and (B) the internal disulfide center (C154–C159). The interatomic distances of 3.0 Å and the clear segregation of electron densities between the sulfur/selenium atoms confirm that these centers are in a reduced, non-bonded state.

We acknowledge the reviewer’s concern about the high reactivity of selenocysteine (Sec) and potential re-formation of the selenylsulfide bond during crystallization. While Sec is indeed reactive, our structural assignments rely on both interatomic distance (> 2.2 Å) and diagnostic electron density shape, not just main-chain conformation, which unambiguously distinguishes the reduced state. Furthermore, refinement and average B-factor analysis confirm uniform thermal motion and no evidence of sample heterogeneity or mixed redox states in the final models. Accordingly, we have incorporated the corresponding PDB IDs into Tables S3, S4, and S6. To improve visual clarity, the transparency of the electron density maps in Figure 1D–G has been enhanced, and the average B-factors for key residues have been supplemented in the respective legends. Furthermore, we have rectified the color scheme by replacing the yellow color for the FAD cofactor and distance indicators to avoid ambiguity. All figure captions involving structural models now include their corresponding PDB IDs.

The supporting information is incomplete and does not provide enough detail to allow the experiments to be reproduced. The proteins were cloned into pET28a, but the location of the His tag (N- or C-terminal) is not specified and needs to be inferred from the structures. The affinity tag does not appear to have been removed, yet its potential impact on enzymatic activity is not addressed. The SECIS element following the GCUG* is also not described. Was it the native *Schistosoma japonicum* SECIS or an *E. coli* formate dehydrogenase SECIS element? The authors note a five–amino acid difference in the SjTrx1i sequence compared to previous reports, but the specific amino acids are not specified. Without a complete description of the amino acid sequences and plasmid constructs, these results could not be reproduced. Overall, a more thorough and detailed Materials and Methods section is required.

We sincerely thank the reviewer for these critical and constructive comments and suggestions.

All recombinant proteins in this study including SjTGR^WT^ (in pABC2a), SjTGR^U597C^ (in pET-28a), and SjTrx1i (in pET-28a) were expressed with an N-terminal His-tag, whereas SjTRP14 (in pET-22b) was expressed with a C‑terminal His‑tag. The tag information for each construct has now been explicitly provided in the Methods section. As the tags are located distal to the enzymatic active sites, we retained them during purification and propose that their impact on protein activity is negligible.

The SECIS (selenocysteine insertion sequence) element used for selenoprotein expression was derived from *E. coli*. This detail, along with appropriate references, has been elaborated in the Methods section to aid reproducibility.

The differences between the amino acid sequence of our SjTrx1i construct and previously reported SjTrx1 sequences are now clarified in the legend of Supplementary Figure S9. The complete amino acid sequences for SjTGR, SjTrx1i, and SjTRP14 have been provided in the Supporting Information to facilitate future studies.

Related to that, error bars and significant figures should be consistent. There are multiple figures where it is not clear whether error bars are not shown because they are missing or very small. For example, panels 1L and 42G, Figure 2G (if it is a representative measurement, add the other two to the supporting information), panels 6A-B, and other panels in the supporting information. Was all data acquired in triplicate? There are no error estimations in Table 1. Overall, the manuscript would benefit from clarification when error bars are too small to be displayed. Lastly, Error analysis should report significant figures consistently. In Biochemistry, we typically keep one or two significant figures. For some measurements, like the ITC, it is hard to imagine that the error can be so precisely estimated as to have three significant figures. For example, line 295 “Kd value of 11.2 ± 3.82 μM” should be rounded to 11.2 ± 3.8 μM.

We thank the reviewer for these meticulous and essential comments regarding data presentation and statistical reporting. We agree that consistency and clarity in error representation are fundamental. We have now systematically addressed each point throughout the manuscript and supplementary information as follows:

1. Clarification on Error Bars (Presence/Absence and Size):

Panels with Missing/Very Small Error Bars: We have carefully re-examined all indicated figures (e.g., Fig. 1L, 2G, 6A-B). For data points where error bars are not visible, we have added an explicit note in the respective figure legends, such as: “(mean ± SD, n=3; In some cases, error bars are smaller than the symbol and thus not visible)”. This clarifies that the errors are present but not graphically resolved.

Representative Measurement: As suggested, the data from the two additional technical replicates for the experiment shown in Figs. 2G, 2H and 4H have been added as two new Supplementary Figures (Fig. S5 and Fig S8), demonstrating the reproducibility of the measurement. And we have added an explicit note “Shown are representative images from triplicate measurements.” in the figure legends.

Replicate Information: All quantitative biochemical and enzymatic assays (including those in Fig. 1L, Table 1, etc.) were performed with a minimum of three independent replicates (n≥3), unless otherwise stated as a single representative trace (now explicitly noted, e.g., for certain ITC thermograms). This statement has been added to the “Statistical Analysis” subsection of the Methods.

2. Addition of Error Estimates to Table 1:

95% confidence intervals for *K*cat (min⁻¹) have now been calculated from the replicates and included in the revised Table 1.

3. Standardization of Significant Figures for Reported Errors:

We have revised all reported errors throughout the manuscript (text, tables, and figure legends) to adhere to the standard practice in Biochemistry: errors are now reported with one or, at most, two significant figures, and the mean value is rounded to the same decimal place.

Specifically, the example cited (Line 295, now Line XXX in the revised manuscript) has been corrected from “11.2 ± 3.82 μM” to “11.2 ± 3.8 μM”.

This rounding has been applied consistently to all ITC-derived parameters, enzymatic constants (Km, kcat), and other quantitative measurements.

We believe these comprehensive revisions have significantly enhanced the rigor, clarity, and consistency of our data presentation. We are grateful to the reviewer for these valuable suggestions.

The error bars for the results in Figure 1 and partial results in Figure 4 were too small to be visualized, and this has been stated in the corresponding figure legends. The other two replicate ITC results for Figure 2G, as well as those for Figure 4H, have been included in the Supporting Information. The errors for *K*cat values have been supplemented in Table 1. Additionally, the *K*d values derived from ITC experiments have been revised.

Reviewer #3: (No Response)

Part III – Minor Issues: Editorial and Data Presentation Modifications

Please use this section for editorial suggestions as well as relatively minor modifications of existing data that would enhance clarity.

Reviewer #1: Minor

Figure 4. The authors utilize various TGR inhibitors in DTNB substrate assays, but report only the structure with auranofin. Please expand on why these other inhibitors are relevant, are the authors implying these inhibitors are of too low affinity to crystallize?

Thank you for your valuable comments. We selected auranofin, TRi-1, and Sj001 to investigate their inhibitory effects on SjTGR enzymatic activity. Auranofin is a well-characterized, irreversible covalent inhibitor of both mammalian thioredoxin reductase (TrxR) and flatworm thioredoxin glutathione reductase (TGR). TRi-1 is a specific, irreversible covalent inhibitor of mammalian TrxR1. In contrast, Sj001 has been determined to be a reversible inhibitor of SjTGR (Fig. S7). This panel of compounds allowed us to compare distinct mechanisms of inhibition (irreversible covalent vs. reversible) targeting the same enzyme.

Despite repeated attempts via both crystal soaking and co-crystallization approaches, we were unable to determine the high-resolution complex structures of SjTGR with TRi-1 or Sj001. The inability to obtain these structures could stem from several technical factors inherent to crystallography (e.g., ligand-induced crystal disorder, conformational heterogeneity, or incompatibility with the crystallization condition) and does not necessarily imply weak binding affinity. This point has been clarified in the revised manuscript to avoid misinterpretation.

Figure 6. Given data showing SjTGR has a higher catalytic rate for reduction of Trxli over TRP14, it is unclear why RNAi assays focused on SjTRP14

We thank the reviewer for this excellent and insightful question, which allows us to clarify the strategic rationale for focusing on SjTRP14 in our *in vivo* studies. While our *in vitro* data confirm that SjTGR reduces both SjTrx1i and SjTRP14, the observed divergent substrate specificity between these two thioredoxin-family proteins prompted our investigation into the unique biological role of SjTRP14.

Specifically, SjTrx1i efficiently reduced classic disulfide substrates like insulin, oxytocin, desmopressin, and somatostatin, whereas SjTRP14 showed no activity toward any of these peptides. This functional distinction suggests that SjTRP14 is not a redundant backup for SjTrx1i but likely serves a specialized, non-overlapping physiological function within the parasite's redox network. The *in vivo* essentiality of such a TrxR-dependent protein with unknown function in schistosomes had not been previously established.

Therefore, our decision to target SjTRP14 via RNAi was driven by a functional genomics approach: to directly test its necessity for parasite survival and reproduction *in vivo*, irrespective of its comparative catalytic efficiency *in vitro*. The severe phenotypes resulting from its knockdown confirm that SjTRP14 performs an indispensable role, validating it as a novel and complementary drug target alongside the well-characterized SjTGR.

Line 59. ‘knowledge’-based design

Corrected as suggested.

Line 89. see PMID 34936384

We thank the reviewer for bringing this important reference to our attention. We acknowledge that our original statement—“The drug target for PZQ is also unknown, which severely hampers the effort to improve its efficacy”—is no longer accurate in light of recent findings.

As the reviewer correctly noted, a transient receptor potential ion channel (TRPM_PZQ_) has been identified as the target of praziquantel (PMID: 34936384). Accordingly, we have removed the outdated statement from the revised manuscript to ensure the accuracy and timeliness of our introduction. We appreciate the reviewer’s diligence in helping us correct this oversight.

Line 154. Reminiscent

Corrected as suggested.

Line 155. Also needs a reference from the literature.

Following the reviewer’s suggestion, we have added the relevant reference in the revised manuscript.

Reviewer #2: From the introduction it is not easy to know which TGR structures are already available. Only one? Consider adding a table t supporting. What is already known about its reaction mechanism? Figure 3S suggests that the introduction can be expanded.

We sincerely thank the reviewer for this constructive feedback. We agree that the original introduction did not adequately inform readers about the current landscape of TGR structural biology and the established knowledge of its catalytic mechanism. To address this, we have compiled a comprehensive overview of all experimentally determined TGR structures deposited in the PDB to date. This information is now presented in a new Supplementary Table S1.

**Table S1. Overview of TGR Structures currently available in the PDB.**

| **Protein name** | **Species** | **PDB ID** | **Reference and main conclusion** |
| --- | --- | --- | --- |
| SmTGR | *Schistosoma mansoni* | 2V6O | SmTGR is comprised of two homo-subunits and each subunit is a structural conjugate of a glutaredoxin (Grx) domain and a TrxR. (PMID: 18300227) |
| SmTGR | *Schistosoma mansoni* | 2X99 | After binding to TGR, NADPH donates electrons from its nicotinamide ring to FAD interacting with TGR with its isoalloxazine ring and the nearby C154/C159 redox couple for transferring the electrons first to the C596/U597 redox couple on the C-terminal segment of the neighboring subunit, and then to the oxidized Trx or the C28/C31 redox couple of the neighboring monomer to reduce GSSG. (PMID: 20659890) |
| SjTGR | *Schistosoma japonicum* | 4LA1 | Only the structure of SjTGR lacking the C-terminal catalytic center has been determined, and no relevant research articles have been published to date. Current literature is limited to reports concerning its protein purification, crystallization, and diffraction data. (PMID: 24419626) |
| EgTGR | *Echinococcus granulosus* | 5W1J | The structural study revealed that TGR functions are achieved not only through a mobile Sec-containing redox center but also by rotation of the Grx domain and distinct binding sites for Grx domain and thioredoxin. (PMID: 28463568) |

The reaction mechanism of TGR as follws: The catalytic cycle of TGR is of a reductive half-reaction and an oxidative half-reaction. After binding to TGR, NADPH donates electrons from its nicotinamide ring to FAD interacting with TGR with its isoalloxazine ring and the nearby C154/C159 redox couple [10, 26] for transferring the electrons first to the C596/U597 redox couple on the C-terminal segment of the neighboring subunit, and then to the oxidized Trx or the C28/C31 redox couple of the neighboring monomer to reduce GSSG. This statement has been included in the Introduction of our original manuscript.

As the reviewer astutely noted, Figure S3 provides important contextual information that was not fully leveraged in the original Introduction. We now explicitly refer to Figure S3 in the revised Introduction as follows: “TrxR1 undergoes sequential reduction by two equivalents of NADPH, proceeding from the fully oxidized state (*E_ox_*) to the two-electron-reduced intermediate (*EH_2_*) with reduced FADH_2_, and finally to the four-electron-reduced enzyme (*EH_4_*), thereby enabling substrate reduction.”.

We are confident that these revisions comprehensively address the reviewer’s concerns and significantly improve the accessibility and scholarly rigor of our manuscript. We are grateful for the reviewer’s guidance in strengthening this work.

In line 204 is 13% change in kcat/km significant enough to call T594 a modulator (see line 206).

We thank the reviewer for this incisive and important comment. We agree that a 13% reduction in catalytic efficiency, while reproducible, does not provide sufficient evidence to support the designation of T594 as a “modulator”—a term that implies a definitive and substantial regulatory role. Accordingly, we have revised the text to adopt more measured and precise language that accurately reflects the magnitude of the observed effect. The original sentence has now been modified to: “...indicating that T594 contributes to maintaining the optimal geometry for electron transfer.” in the revised manuscript.

Line 273 “However, its structure basis of its function remained elusive.”

There is a structure of auranofin (Au) with TrxR so it is more clear to state that -

However, its structure basis of its interactions with TGR is unknown.

Thank you for your suggestion. We have accordingly modified the sentence in the revised manuscript.

Line 329 about oxytocin conflicts with observations reported in reference 32 (line 322). Please comment on the differences between the activity assays here and in reference 32.

Thank you for your comments.

The original assays described in Ref. [32] were performed in a 200 µL system containing 50 mM HEPES (pH 7.0), 1 mM EDTA, 200 µM NADPH, 50 nM TrxR1, 50 µM TRP14 or Trx1, and 0.1 mM oxytocin.

In our study, the reaction was scaled to a 100 µL mixture with modified component concentrations: 100 mM HEPES (pH 7.0), 1 mM EDTA, 600 µM NADPH, 50 nM SjTGR, 5 µM SjTrx1i or SjTRP14, and 1 mM oxytocin. Notably, we employed higher concentrations of both NADPH and the substrate oxytocin to ensure robust detection of activity.

Beyond oxytocin, we further evaluated the substrate specificity of SjTRP14 using desmopressin and somatostatin. Under the assay conditions described, SjTRP14 showed no detectable reductive activity toward any of these three disulfide-containing peptide substrates.

Line 504 a reference describing C321.ΔA is needed.

As suggested, we have included the relevant reference describing C321.ΔA in the revised manuscript.

Line 548-549 “To determine the reduced structures of SjTrx1i and SjTRP14, the crystals were soaked in 5 mM DTT for 10 minutes” and immediately frozen?

We thank the reviewer for seeking clarification on this important experimental detail. The reviewer is correct in identifying a point that required more precise description.

Yes, following the 10-minute soak in the 5 mM DTT solution, the crystals were immediately harvested, cryo-protected, and flash-frozen in liquid nitrogen for data collection. The original sentence was intended to convey this sequence but omitted the intermediate harvesting step. We have revised the text in the Methods section for clarity and completeness.

For Fig 1L the caption describes “performed using DTNB as the substrate, measuring TNB production at 421 nm” but figure lists 412 nm. Which wavelength was used?

We sincerely thank the reviewer for identifying this inconsistency. The DTNB reductase activity assay shown in Fig. 1L was monitored at 412 nm, not 421 nm as previously stated. We have now corrected the wavelength description in the caption of Fig. 1L in the revised manuscript. We apologize for this oversight and appreciate the reviewer's careful reading.

Supporting:

Clarify that Figure S1B-E is exclusively describing SjTGR U597C.

Provide similar information for the wild-type SjTGR.

Thank you for your suggestion. We have clarified that Figures S1 B–E describe only SjTGR^U597C^ and supplemented the relevant data for wild-type SjTGR in Figure S1.

Add PDB ID to captions

As suggested, relevant PDB IDs have been added to the main text, figure captions and supplementary tables in the revised manuscript.

Figure S6, clarify that these are three independent repeats and that error bars are too small to be observed?

We thank the reviewer for this helpful suggestion. We have clarified these are three independent repeats and noted in the figure caption that some error bars are smaller than the symbol and thus not visible.

Typos:

Unclear sentences:

Sentence 115 consider changing “deciphering the mechanisms of TGR’s catalysis” to in depth studies of TGR’s reaction mechanism.

Corrected as suggested.

Line 153 instead of mutant use variant

A change in nucleic acid is a mutant

A change in amino acid is a variant

Also use the term selenylsulfide bond for Se-S

Corrected as suggested.

Line 188 introduce abbreviation Rattus norvegicus TrxR as RnTrxR1. Same for BmTrxR1 line 217. Introducing the abbreviation in figure 1 caption is not sufficiently clear.

Thank you for your comments and suggestions. Accordingly, we have introduced abbreviation *Rattus norvegicus* TrxR1 as RnTrxR1, *Brugia malayi* TrxR1 as BmTrxR1 in the revised manuscript. We have also revised the legend of Fig. 1K to accurately clarify the abbreviation included: “Sequence alignment of the C-terminal redox motif from structurally characterized TGR and TrxR homologs including *Homo sapiens* TrxR1 (hTrxR1, NCBI accession: NP_877393.1), *Rattus norvegicus* TrxR1 (RnTrxR1, NCBI accession: O89049.5), *Schistosoma mansoni* TGR (SmTGR, NCBI accession: XP_018649018.1), and *Schistosoma japonicum* TGR (SjTGR, NCBI accession: ACH86016.1).”

Line 196 substitute was with is?

Corrected as suggested.

Line 201 substitute “was with” with exhibited

Corrected as suggested.

Line 209 “The complex structure of SjTGR and NADPH” consider simplifying to The structure of SjTGR complexed to NADPH

Corrected as suggested.

Line 322 Although hTrx1 can reduce insulin, hTRP14 cannot, although both can reduce both oxytocin and vasopressin.

Corrected as suggested.

Consider more targeted keywords.

We thank the reviewer for this suggestion. Accordingly, we have added “selenocysteine” and “drug target” to the keywords list in the revised manuscript.

Line 336 “These results suggested that SjTrx1i and SjTRP14 were for different substrates.” These results suggested that SjTrx1i and SjTRP14 have different substrate specificity.

Corrected as suggested.

Reviewer #3: Fig S1 - Authors need to define the column names on their gel in panel B (what are FT, W1, W2, etc?).

We thank the reviewer for pointing out the omission of lane annotations in the gel figures. As suggested, we have now defined all relevant abbreviations in the legends of Fig. S1 and Fig. S9 as follows: M: marker, S: supernatant, P: precipitate, FT: flow-through, W1–4: wash fractions 1 to 4, E: eluate.

Fig S2 - the use of Figure panel labels "A" and "B" within panel 2A is this figure is a little confusing. Suggest using roman numerals for the subpanels or just referring to them as left vs right in the figure legend. The author also haven't used this format consistently among the panels within this figure or between figures. I suspect this is included in error, but please check.

We thank the reviewer for this helpful reminder. To ensure consistent subunit annotation across all figures, we have now revised Fig. S2A to label the two subunits as FAD(A) and FAD(B). This adjustment aligns with the uniform A/B panel notation adopted throughout the manuscript, including the residue-based subunit designation in Fig. 4A–C (e.g., C154(A), C154(B)).

Line 173-174 "which was crucial for maintaining TGR functionality in parasitic flatworms." - is this statement relating to the results of this current study or prior literature? If the former, please explain how this was determine. If the latter, please provide citations to support the statement.

We thank the reviewer for identifying this lack of clarity. The statement in question was intended to refer to prior literature, not to findings from the present study. However, we recognize that the original phrasing was ambiguous and failed to provide appropriate citations. Accordingly, we have added the corresponding citations to support the statement in the revised manuscript.

Lines 196-197 - the authors reference a glycine residue in other platyhelminths in Fig 1K, but this data is not shown in the alignment. Is this informatin from the literature? Please include it.

We thank the reviewer for pointing out this inaccuracy in our statement. We acknowledge that our original wording was imprecise and could mislead readers into believing that a broad multi-species alignment of platyhelminth TGR/TrxR sequences had been performed.

In fact, the alignment presented in Fig. 1K includes only the TGR and TrxR sequences from species whose C-terminal structures have been experimentally determined and are currently available in the PDB database (hTrxR1, RnTrxR1, SmTGR and SjTGR). The reference to “other platyhelminths” was an overstatement and has now been corrected.

To address this issue, we have:

Revised the legend of Fig. 1K to accurately reflect the sequences included: “Sequence alignment of the C-terminal redox motif from structurally characterized TGR and TrxR homologs including *Homo sapiens* TrxR1 (hTrxR1, NCBI accession: NP_877393.1), *Rattus norvegicus* TrxR1 (RnTrxR1, NCBI accession: O89049.5), *Schistosoma mansoni* TGR (SmTGR, NCBI accession: XP_018649018.1), and *Schistosoma japonicum* TGR (SjTGR, NCBI accession: ACH86016.1).” And also revised the Fig. S1A legend accordingly.

Corrected the main text accordingly, removing the unsupported claim and specifying that the comparison is limited to the sequences shown in the alignment.

Lines 200-202 - The wording is a little confusing here. Are the authors stating that their data showed the dfference in DNTB reducatase activity between the wild-type and mutated form or that the structure analyses predict that this should be the case? I presume the former given the figure panels, but the statement 'would abolish' is a bit ambiguous.

We thank the reviewer for highlighting this ambiguity in our wording. We confirm that the conclusion regarding the essential role of the selenol group in SjTGR catalysis is drawn exclusively from direct experimental measurements, not from structural inference.

As shown in Fig. 1L, the SjTGR^U597C^ with a substitution of the critical selenol group completely lost redox catalytic activity in our DTNB reductase assays, while the wild-type enzyme exhibited robust activity. This functional loss provides unambiguous evidence that the selenocysteine residue is indispensable for catalysis.

To eliminate any potential misinterpretation, we have revised the text in the revised manuscript as follows: “Further enzymatic investigation showed that the wild-type enzyme exhibited a robust DTNB reductase activity, and SjTGR^U597C^ with a mutation on the critical selenol group had no detectable redox catalytic activity under the same assay conditions (Fig. 1L), demonstrating that the selenol group is essential for SjTGR enzymatic function.”.

Fig 2C and elsewhere - the use of yellow lettering over the white background of the figure is quite difficult to see. Maybe a darker yellow shade or another darker colour would be better?

We thank the reviewer for this helpful observation. As suggested, we have rectified the color scheme by replacing the yellow color for the FAD cofactor and distance indicators with darker colour to avoid ambiguity and improve visual clarity. These revisions have been applied consistently across all relevant figure panels.

Lines 271-273 - I presume this statement is related to prior literature. Please cite the relevant studies presuming that is the case.

We thank the reviewer for this suggestion. As recommended, we have now cited Reference 18 to support the corresponding statement in the revised manuscript. The citation has been placed at the appropriate location to substantiate the claim and provide readers with the relevant primary source.

Fig 4 - As above, the user of 'A' and 'B' sublabels within some figure panels but not others and the similar use of 'A' and 'B' for different panels within the figure as a whole is a bit confusing. I suggest either denoting left vs right in the figure legend or using another sublabel, but then using it consistently. For example 'A' and 'B' are used to differentiate the left vs right figures in panels 4A, B, C, but not 4D.

We thank the reviewer for pointing out the inconsistency in subunit labeling across our figure panels. We apologize for the confusion caused by the use of internal “A” and “B” sublabels within the images.

To address this issue and ensure consistent subunit annotation throughout the manuscript, we have removed the independent “A” and “B” indicators within Fig. 4A–C. Following the convention now adopted in the revised Fig. S2A (where FAD molecules are annotated as FAD(A) and FAD(B) for the two subunits), we now denote the two subunits in Fig. 4A–C by explicitly annotating residues with their corresponding subunit designation—for example, C154(A) and C154(B).

Fig S7- As above the column lables in Fig S7A and E need to be defined.

As suggested, we have now defined all relevant abbreviations in the legends of Fig. S7A as follows: M: marker, S: supernatant, P: precipitate, FT: flow-through, W1–4: wash fractions 1 to 4, E: eluate.

Fig 5 - what do the authors make of the observation that mutating T594, which the authors note (lines 197-198) provides a unique contribution to the conformation of SjTGR and the t594V mutation altered the catalytic effiency of SjTGR (lines 204/205), doesn't affect insulin reduction? Is this expected? The authors show the binding data for T594V in figure 5B, but do not discuss it.

We thank the reviewer for this incisive and important observation. The reviewer correctly notes an apparent discrepancy: the T594V mutation reduces the catalytic efficiency of SjTGR against its standard substrate DTNB (Fig. 1L–M), yet it does not impair the enzyme's ability to support insulin reduction by SjTrx1i (Fig. 5B). We agree that this point warrants explicit discussion, and we have now addressed it in the revised manuscript.

We propose that this differential effect reflects substrate-dependent conformational requirements within the C-terminal redox relay mechanism of SjTGR. DTNB is a direct, small-molecule substrate that accesses the C-terminal selenothiol motif directly. The hook-shaped conformation stabilized by T594 appears to be critical for optimizing the geometry of electron transfer to this small, diffusible substrate. Disrupting this conformation (T594V) therefore measurably impairs DTNB reductase activity. Insulin reduction, by contrast, is an indirect, protein-mediated process. It requires electron transfer from SjTGR to SjTrx1i, which then reduces insulin. This multi-step relay involves protein–protein recognition and interdomain electron transfer, which may be less sensitive to subtle geometric perturbations at the very C-terminus. The T594V mutation, while affecting the local conformation, does not appear to globally disrupt the enzyme's ability to engage and reduce its physiological protein substrate, SjTrx1i.

We have added the following paragraph to the Discussion section to explicitly address this point: “Furthermore, although the T594V mutation reduced the catalytic efficiency of SjTGR toward the small-molecule substrate DTNB by approximately 13%, it did not impair insulin reduction in the coupled Trx1 system (Fig. 5B). This divergence likely reflects distinct conformational requirements for direct substrate turnover versus protein-mediated electron transfer. The hook-shaped C-terminal conformation stabilized by T594 appears to be more critical for optimizing the geometry of direct selenothiol–substrate interactions than for maintaining productive engagement with the physiological redox partner SjTrx1i.”.

Fig 5J the black text of the magenta protein skeleton is quite difficult to read. Maybe white text where there is an overlap, or arrange the labels outside the protein model with dash lines?

We thank the reviewer for this helpful observation regarding the readability of Fig. 5J. We agree that the black text over the magenta protein model caused legibility issues, particularly in regions of color overlap.

To address this, we have replaced the original magenta protein skeleton with a lighter shade to improve contrast. The revised figure now offers significantly improved clarity and accessibility.

Overall comment - the paper would benefit from a little more context around so of the data included in some figure panels vs data left out or maybe not gathered. For example, there may be a perfectly reasonable explanation for why the authors present the binding affintity of Sj001 to SjTGR in Figure 4H but not similar binding affinity data for Auranofin or TRi-1, but they don't provide any context for this decision. Similarly, are the mutants included in Fig 5B different from the ones included in 5C. Both panels look at the capactiy fo SjTGR mutants to reduce SjTrk1i, but the latter examined behaviour under DTT conditions. Again, there may be an clearl explanation, but the authors don't appear to provide it. In another example. why were C41S and C44S mutants included in 5H? Both, why these mutations in 5H, and why not these mutations in 5E-G? Again, there may likely be a clear reason for this, but some context needs to be provided.

Thank you for this constructive comment regarding the contextualization of our experimental design and data inclusion/exclusion. We greatly appreciate the opportunity to clarify the rationale behind these choices, and have incorporated corresponding explanatory details into the relevant figure legends and main text in the revised manuscript.

Regarding the binding affinity data presented in Figure 4H, Auranofin and TRi-1 are well-characterized irreversible covalent inhibitors of TGR (PMID: 29444979) that form non-dissociable covalent linkages with the enzyme’s active site. As standard binding affinity assays rely on reversible association–dissociation equilibria, reliable quantification of their binding affinities for SjTGR was technically unfeasible. In contrast, Sj001 acts as a reversible inhibitor of SjTGR (Figure S7C), enabling valid measurement of its equilibrium binding affinity—this is why only Sj001 binding data are presented in this panel. We have added a statement to clarify this explanation in the Discussion section of the revised manuscript.

For the mutant activity panels in Figure 5, Figure 5B was designed to assess the ability of different SjTGR mutants to directly reduce SjTrx1i. Figures 5C and 5D address a distinct experimental aim, focusing on how DTT treatment or co-incubation with SjTGR influences the insulin-reducing activity of SjTrx1i, its variants, and SjTRP14, rather than evaluating direct reduction of SjTrx1i by SjTGR mutants. We have provided a statement to explicitly address this point, and the added text reads as follows: “It could be shown that SjTrx1i could reduce insulin with both electron donors, but SjTRP14 could not (Fig. 5C-D), indicating that SjTRP14 exhibited no insulin-reducing activity, either with DTT or when coupled with the SjTGR enzymatic reducing system.”.

Concerning the inclusion of SjTRP14^C41S^ and SjTRP14^C44S^ exclusively in Figure 5H, wild-type SjTRP14 exhibited no detectable catalytic activity in the assays shown in Figures 5E–5G. We therefore omitted these two cysteine mutants from these panels, as no functional readout was available to assess mutational effects. In contrast, wild-type SjTRP14 displayed clear peroxidase activity toward hydrogen peroxide in Figure 5H, prompting us to include these mutants to determine whether the Cys41 and Cys44 residues are essential for this catalytic function. We have provided a clear explanation in the Discussion section of the revised manuscript.

Lines 365-383 - are the phenotypic outcomes identified through SjTGR or TRP14 silencing similar to the effects seen with the application of auranofin or other inhbitors? Has this been examined in previous studies?

We thank the reviewer for raising this highly relevant and insightful question, which bridges our genetic knockdown findings with the established pharmacology of schistosome redox biology. The reviewer is correct to note the conceptual parallel. Indeed, the phenotypic consequences we observed following RNAi-mediated silencing of SjTGR—specifically, the significant reduction in worm burden, impaired egg production, and associated tissue pathology in mice—are consistent with, and effectively phenocopy, the major therapeutic outcomes reported for pharmacologic inhibition of TGR.

As the reviewer likely knows, auranofin is a well-characterized gold-containing inhibitor that potently targets thioredoxin glutathione reductase (TGR). Previous *in vivo* studies (e.g., PMID: 17579510, PMID: 22384025) have demonstrated that auranofin treatment leads to marked worm burden reduction and suppression of egg laying in murine models of schistosomiasis, mirroring our genetic knockdown results. Similar anti-schistosomal phenotypes (reduced motility, decreased egg output, and ultimately death) have also been reported for other chemical classes of TGR inhibitors in both *in vitro* and *in vivo* settings (PMID: 37349300; PMID: 33902686).

Regarding TRP14, the situation is different and highlights a novel aspect of our work. To the best of our knowledge, no specific, potent inhibitors of schistosome TRP14 have been developed or reported in previous studies. While the broader thioredoxin system is recognized as a drug target, most inhibitor development has focused on TGR or TrxR. Thus, the severe worm reduction and oviposition defects we observed upon SjTRP14 knockdown represent a novel phenotypic validation of this protein as a potential drug target. Our findings suggest that TRP14 is equally critical for parasite survival and reproduction, thereby identifying it as a promising new candidate for future inhibitor discovery.

We have added a paragraph in the Discussion section that synthesizes these points: "The severe phenotypes resulting from SjTGR knockdown including reduced worm burden and suppressed fecundity, closely mirror the established anti-schistosomal effects of pharmacological TGR inhibitors, such as auranofin [17-18, 20] and other inhibitors [10, 21-23, 31]. This functional congruence strongly reinforces TGR as a critical and druggable hub within the parasite's redox defense system. In contrast, the similarly profound impairment caused by SjTRP14 silencing has not, to our knowledge, been previously recapitulated by chemical inhibition, as specific TRP14 inhibitors remain undeveloped. Our study therefore provides the first direct genetic evidence that TRP14 is essential for *S. japonicum* survival and reproduction *in vivo*, nominating it as a compelling and novel target for future therapeutic discovery aimed at disrupting the parasite's thioredoxin pathway."

We hope this clarification and the corresponding manuscript revision adequately address the reviewer's excellent question.

Fig 6d - what is meant by 'adult insects' in the figure legend for 6D. I assume this is a typo.

We thank the reviewer for noting the typo in Figure 6D. The y-axis label has been corrected to “Number of adult worms” in the revised figure, which has been replaced in the resubmission. This correction does not affect any of the study's conclusions. We apologize for the oversight.

Fig 6 - What are the error bars in the various bar charts in this figure? Standard Deviation, Standard Error? Or something else? Please specify in the figure legend.

Thank you raising this critical issue. In this study, experimental data were presented as mean ± standard deviation (SD) for at least three independent experiments. These methodological details have been explicitly added to the relevant Methods section and figure legends in the revised manuscript.

In addition to the revisions described above, we have also

Updated the PDB IDs in the relevant sections of the revised manuscript to reflect the final deposition of our structural data.

Revised the format of several references throughout the manuscript to comply with the journal's specific requirements.

Updated the Funding statement in the revised manuscript to include an additional supporting grant that contributes toward the Article Processing Fee (APC). The revised statement now reads: “This work was supported by grants from the National Natural Science Foundation of China (nos. 82102428 to S.H.), the Natural Science Foundation of Hunan Province (nos. 2022JJ40663 to S.H.), and the Open Research Project of the Regional Collaboration Centre for Schistosomiasis Control Technology in Lake Regions for the Year 2025-2026 (nos. 20254003 to S.H.). The funders had no role in study design, data collection and analysis, decision to publish, or preparation of the manuscript.”

Removed the duplicate image from the revised manuscript. Specifically, because the total number of collected worms has been fully represented in Fig. 6D, the redundant image previously included in Fig. S10 of original submission has been deleted from the Supporting Information. Correspondingly, the figure numbering in the Supporting Information has been updated; the former Fig. S10 now corresponds to Fig. S12 in the revised manuscript.

Adjusted the format of the author affiliations to comply with journal style.
